# Attitudes of clients of Dutch pest controllers towards animal welfare in the management of liminal rodents

**DOI:** 10.1017/awf.2023.35

**Published:** 2023-05-26

**Authors:** Maite AAM van Gerwen, T Bas Rodenburg, Saskia S Arndt, Bastiaan G Meerburg, Franck LB Meijboom

**Affiliations:** 1 Centre for Sustainable Animal Stewardship, Division of Animals in Science and Society, Department Population Health Sciences, Faculty of Veterinary Medicine, Utrecht University, Yalelaan 2, 3584 CM Utrecht, The Netherlands; 2 Division of Animals in Science and Society, Department Population Health Sciences, Faculty of Veterinary Medicine, Utrecht University, Yalelaan 2, 3584 CM Utrecht, The Netherlands; 3 Wageningen Livestock Research, Wageningen University & Research, De Elst 1, 6708 WD Wageningen, The Netherlands; 4 Dutch Pest & Wildlife Expertise Centre (Stichting Kennis- en Adviescentrum Dierplagen, KAD), Nudepark 145, 6702 DZ Wageningen, The Netherlands

**Keywords:** Animal welfare, mice, pest control, rats, rodent control, rodent management

## Abstract

Rodent control tends to involve methods that cause animal suffering, but little attention has been paid to the animal welfare implications of rodent control. The aim of the current study was to gain insight into the opinions and attitudes of clients of Dutch pest controllers, regarding liminal rodents, rodent control, and rodent welfare. A better understanding of their attitudes may contribute to more ethical rodent management programmes. An online survey among 248 clients of Dutch pest controllers was carried out. Respondents, especially those within the agricultural sector, have a relatively negative attitude towards rats and mice. Respondents in the agricultural subgroup do not consider the welfare of liminal rodents important. They also think that the welfare impact of commonly used control methods is limited, and they have low tolerance levels for the presence of rodents. Respondents from other sectors have a far more positive attitude towards rats and mice, consider their welfare to be of greater importance, have a greater estimation of the welfare impact of control methods and show greater tolerance levels towards rodents. The respondents from the latter subgroup have a similar attitude compared to Dutch pest controllers participating in a previous survey. The findings of the current study firstly provide useful information for the further development and practical implementation of preventive control methods. Secondly, they provide input for a more animal-friendly rodent control and for the development of an assessment framework to support ethical decision-making. Finally, they can be helpful for further research and the communication and co-operation between professional pest controllers and their clients.

## Introduction

Commensal Norway and black rats (*Rattus norvegicus* and *R. rattus*) and house mice (*Mus musculus*) are killed in large numbers globally because they cause different forms of nuisance and are considered pest animals. Nuisance can take various forms, including damage to human property, consumption or contamination of food and feed, spread of disease or simply being an unwanted presence. Rodents, that can be considered liminal animals, are often stigmatised as aliens or invaders that do not belong in human societies (Donaldson & Kymlicka 2011). Liminal animals are non-human animals that are neither wild, nor domesticated, and live their lives amidst humans between nature and culture (Donaldson & Kymlicka 2011). Commensal rats, house mice and pigeons are examples of animals that are considered liminal by Donaldson and Kymlicka (2011).

Little attention tends to be paid to the welfare and moral status of liminal rodents and this topic remains is often largely neglected in the practice of rodent management (Van Gerwen & Meijboom 2018). Meanwhile such controls as coagulant rodenticides, cholecalciferol and glue traps are commonly used which have a severe or even an extreme impact on animal welfare (Broom 1999; Mason & Littin 2003; Meerburg *et al*. 2008; Baker *et al.* 2022; Krijger *et al.* 2022; De Ruyver *et al*. 2023). Recently in The Netherlands different (policy) developments have been implemented that may contribute to a more animal-friendly approach to rodent management (Van Gerwen & Meijboom 2022). One being that rodent management should be performed in accordance with professional standards based on the principles of Integrated Pest Management (IPM). These define rodent management as consisting of several phases: identification of possible pest species; determination of the threshold level; prevention; monitoring; and control (for reviews, see Meerburg *et al.* 2008; Van Gerwen & Meijboom 2022).

Also, at a European level and in both Flanders and the UK, recent developments may contribute to a more animal-friendly approach to rodent control. Firstly, in 2021, the development of European norms for snap traps was instigated (Schlötelburg *et al.* 2021). Mechanical snap traps have been found to range widely in their welfare impact (varying from zero to extreme) (Baker *et al.* 2022; De Ruyver *et al*. 2023). According to Baker *et al.* (2022) snap traps should be regulated and tested to ensure rapid unconsciousness and death. Correct trap placement and use are also important aspects to be considered. The German Environment Agency (Umwelt Bundesamt) works on behalf of the European Commission on the development of a certification system for rodent snap traps. As part of this system, a guidance document for the evaluation of break back/snap traps has already been published (Schlötelburg *et al.* 2021).

Secondly, since April 2022 England and Wales have the Glue Traps (Offences) Act 2022 in force, which has been introduced in the UK Parliament following scientific findings (UK Government 2022). The Act bans the use of glue traps to catch rodents for persons without a licence. Scotland is proposing to introduce a ban on the use of glue traps (Scottish Government 2022). In several other countries, including New Zealand, the Republic of Ireland and India the use of glue traps on rodents has already been prohibited (Universities Federation for Animal Welfare [UFAW] 2022).

Finally, in 2022 and 2023, two studies (Baker *et al.* 2022; De Ruyver *et al.* 2023) assessing the welfare impact of commonly used rodent control methods were published. Both studies showed, by means of a welfare impact assessment, that most commonly used rodent control methods have a significant impact on rodent welfare. They also highlight uncertainties in the welfare impact of, for example, snap traps. The results from the study of De Ruyver *et al.* (2023) were also used by the Flemish Animal Welfare Department to inform the public about animal welfare in rodent control via a website and information brochures.

In an earlier study we found that Dutch stakeholders involved in rodent management believed the future control of rats and mice could be more animal-friendly than is currently seen (Van Gerwen & Meijboom 2018). For example, by applying more preventive measures and paying more attention to animal welfare aspects of control methods. In a survey among Dutch pest controllers (Van Gerwen *et al.* 2020) it was revealed that respondents felt the welfare of liminal rodents mattered. They do, however, deem the welfare of pest rodents to be less important that of laboratory, farm, companion and wild animals other than rats and mice. When asked, the respondents evaluated existing control methods differently in terms of their welfare impact. Glue traps and drowning were estimated to have a very high negative impact on welfare, rodenticides a high negative impact and snap traps, shooting (rats) and preventive methods a very low or non-existant impact on welfare. The weight that respondents attributed to the interests (specified as living, freedom and welfare) of liminal rodents depended on the type of the real-life situation (mice in a hospital vs. rats in a ditch). The terms ‘interests of animals’ or ‘animal interests’ are used here to refer to issues that are of importance to the animals themselves and includes staying alive (living), having the freedom to roam and make own decisions (freedom) and maximising their own welfare (welfare). Respondents from the previous study considered prevention of nuisance an important, effective, and animal-friendly method of controlling rodent nuisance. However, they indicated that clients who are engaging a pest control contractor do not always invest sufficient time and money in preventive methods. Almost half of the participating pest controllers in the survey indicated that they experienced problems when weighing rodent interests against those of humans (e.g. financial costs, food safety, hygiene) in practice. The majority (64.9%) of the problems mentioned by respondents from the former study were client-related and had to do with clients that lacked the willingness to invest sufficient money in preventive methods. According to the respondents, changes in client awareness, improved knowledge and willingness to invest in prevention are important factors to improve the implementation of preventive methods and safeguard animal welfare. There may be a mismatch between the views and expectations of professionals and their clients as revealed by different studies comparing expectations of professionals and their clients (e.g. patients and customers) in other sectors (human health care and veterinary practice) (Demetriou *et al*. 2009; Poost-Foroosh *et al.* 2015; Sladdin *et al.* 2019). But professional pest controllers may also underestimate the actual knowledge of their (potential) clients. A study by Burt and Lipman (2021) showed the responses of members of the Dutch general public to statements about IPM were correctly predicted by Dutch pest controllers in only two of the 14 statements. The study shows that members of the public have a reasonable level of knowledge about IPM and prevention. It also shows that professional Dutch pest controllers underestimate the knowledge of members of the public, especially when it comes to preventive measures such as preventing rodents from entering buildings and taking away food resources. Investigating and acknowledging the discrepancies in views and knowledge between professionals and clients might facilitate better communication between professional pest controllers and their clients, joint decision-making and help ensure a better application of preventive methods and a more animal-friendly rodent control.

The aim of the current study was to gain insight into the opinions and attitudes of clients of professional pest controllers (e.g. organisations and companies that engage a pest control contractor), regarding liminal rodents, rodent control, and rodent welfare. This study is part of a larger body of work that also looked at client attitudes towards IPM and the application of preventive measures (Van Gerwen *et al.* in prep). The complete study serves: (1) to verify the findings of the previous study among pest controllers (Van Gerwen *et al.* 2020); and (2) explore whether the ideas of the pest controllers match client views. Furthermore, (3) the outcomes will be used to develop an assessment framework that can support ethical decision-making in the practice of rodent control and be used by both pest controller and client. We consider the input of pest controllers and clients to be important for the development and practical implementation of such a framework. Insight into opinions and attitudes of both pest controller and client may in this way contribute to a more ethical rodent management where the moral position of rodents and their welfare is subject to greater care and consideration.

## Materials and methods

### Survey

Data were collected via means of an online survey among professional clients of Dutch pest controllers. Professional clients can be affiliated to different sectors, including municipalities, food-processing companies, healthcare services, restaurants and hotels, zoos, and the agricultural sector (type of company within the agricultural sector was not further specified, but besides farms other companies in the supply chain were included). The survey was set up in Dutch using Qualtrics software (Qualtrics, Provo, UT, USA). Answers were translated into English for the purposes of this article. The link to the survey, together with the call to take part, were placed on the website of Utrecht University and disseminated throughout The Netherlands via professional associations (newsletters and websites), personal networks and social media. It was explicitly stated in the invitation that respondents should be the persons overseeing rodent management and/or contacts with the pest controller.

No sample size was predefined since the main function of the survey was to provide a descriptive overview of views and opinions. The survey remained open between February 25th and March 27th, 2020 and the average time taken for completion was 27 min. Results were obtained and processed anonymously.

Questions (32 in total) were based on, and largely equal to, an earlier survey among pest controllers (Van Gerwen *et al.* 2020) enabling valid comparisons to be made between the two respondent groups (see *Discussion*).

The questions differed in form, consisting of five-point Likert-style questions (never, seldom, sometimes, often, always), continuous rating scales from 1 (e.g. totally disagree or not important) to 10 (e.g. totally agree or very important) using slider bars, open, multiple-choice, and multiple response questions. A range from 1 to 10 was used for the rating scales since this is a familiar to Dutch people due to its use for grading at schools. For certain questions, respondents were able to provide additional information or supplementary answers not provided in the lists. At the beginning of the survey, rats and mice were specified as black and brown rats and house mice.

The complete online survey consisted of eight sections, divided into a part (a) with animal welfare-related questions and subsequent part (b) showing questions related to IPM and prevention of rat and mouse infestations. Both parts of the survey resulted in many relevant data. Therefore, in the present study, we focus on the animal welfare aspects of rodent management and only the data from part (a) were used (sections one to four and part of the data from section seven of the survey). The other results, containing data regarding IPM and prevention, will be published in a separate paper (Van Gerwen *et al.* in prep).

The relevant survey sections discussed here are as follows:

**Section 1**: General questions about the company respondents work for and their positions.

**Section 2**: Questions about hiring a professional pest controller and for what reasons and purpose. Based on answers of two questions in section two, respondents were selected for continuation of the survey. Only respondents working for a company that hired a professional pest controller for rodent control were selected. This was almost 61% of the total participants who started the survey (see also *Results*).

**Section 3**: Five statements/propositions about the general perception of rats and mice, where respondents could agree with or not on a 1 (totally disagree) to 10 (totally agree) continuous rating scale.

**Section 4**: Questions regarding conceptions of animal welfare (multiple-response questions), the importance of rodent welfare (Likert scales and 1 to 10 rating scales), responsibilities for animal welfare and the impact of ten control methods in terms of animal suffering on a 1 (no welfare impact) to 10 (very large welfare impact) continuous rating scale. In this section respondents were also asked about the relative importance of animal welfare for five different animal categories/contexts, namely rodents as pest animals, wild animals other than rats and mice, laboratory animals, farm animals and companion animals.

**Part of section 7**: Questions about the weight of animal interests in 12 different real-life scenarios on a 1 (animal interests do not count) to 10 (animal interests count heavily) continuous rating scale and the experience of problems when weighing human and animal interests. Animal interests were defined as ‘living, freedom, and welfare.’

At the end of the survey (section 8), data relating to respondent demographics were collected, namely gender, age, level of education and pet ownership.

### Statistical analysis

Analysis of the survey results was carried out using the IBM^®^ SPSS^®^ Statistics for Mac (Version 26) computer programme (IBM Corp, Armonk, NY, USA), using descriptive statistics and inferential statistics for the rating scale data.

The general questions about ‘pest control’ were analysed for all respondents who started the survey (n = 248). Respondent demographics could only be analysed for respondents who fully completed the survey (n = 108), since these questions were asked at the end. Other questions were analysed for the group of respondents with a professional pest controller for the control of rodents only. Incomplete surveys were also included and therefore the number of respondents included in the analyses could vary between 108 and 151 depending on the specific question.

Since the number of respondents from the agricultural sector was relatively large (105 out of 151 or 73 out of 108) compared to other professional sectors, a new grouping variable ‘subgroup’ with two categories (*subgroup agri* and *subgroup other*) was created. Furthermore, to ensure that any statistical findings were not wrongly attributed to the large group of respondents from the agricultural sector, analyses were also performed separately within both the respondents from *subgroup agri* and from *subgroup other.*

As, for some age categories, the number of respondents was very low (only one or even zero), four new age categories were created (younger than or equal to 30 years, 31 to 40 years, 41 to 50 years and 51 or older). The original age categories (six in total) also included the categories younger than 21 years, 21 to 30 years and older than 67 years. To analyse education level the six original categories were merged into two (practical vs. theoretical education). Practical education included the following education groups: high school and basic vocational education while theoretical education included university of applied sciences bachelor, university bachelor, university master and doctorate/PhD.

For the rating scale data, normality tests were performed using the Shapiro-Wilk and Kolmogorov-Smirnov tests. Since most of the data (except for some rating scales within subset rest) were not normally distributed, non-parametric analyses were performed for all data.

The Friedman repeated measures test (*omnibus*) and the Wilcoxon matched-pairs signed-ranks test (*post hoc*) were used to test for differences between dependent variables (e.g. ‘importance of animal welfare’, ‘scored welfare impact of different control methods’ and ‘weight of animal interests in different practical scenarios’). The Kruskal-Wallis test (*omnibus*) and Wilcoxon Mann-Whitney test (*post hoc*) were used for testing differences between independent grouping variables (factors). The following grouping variables were tested: (1) *subgroup agri* or *subgroup other*; (2) gender; (3) ownership of companion or hobby animals; (4) age class; and (5) education level. Descriptive statistics of continuous rating data (on a 1 to 10 scale) were provided as medians with inter-quartile ranges (IQR, displayed as Tukey’s Hinges Q3–Q1). These data were displayed graphically as boxplots.

For the *omnibus* and *post hoc* tests, Monte Carlo (number of samples was 10,000) and exact *P-*values (two-tailed) were, respectively, calculated. To compensate for the increased chance of a type I error due to multiple hypotheses testing, values of *alpha* (*α*) were adjusted with the Dunn-Šidák correction. The adjusted *alpha* values for each test used can be found in Supplementary Tables S1 and S2. Statistically significant *P-*values are marked with an asterisk (*) and are reported to six (e.g. *P* = 0.000414*) or seven (*P* < 0.0000005*) decimal places. This number of decimal places was used in order to be able to compare the *P-*values with the corrected values of *alpha* which have a similar number of decimal places.

Statistical significance represented by *P-*values may not necessarily confirm practical importance. Therefore, besides *P-*values, estimated effect sizes were calculated. In Van Gerwen *et al.* (2020) the formulae for the correction of *alpha* values and thresholds for interpretation of effect sizes can be found.

### Ethical approval

The survey research reported in this article involves healthy human participants and does not utilise any invasive subjects, techniques, substance administration or psychological manipulations. Besides age, education level and pet ownership, the survey did not contain personal or sensitive information. We recruited participants through newsletters and websites and included the link to the questionnaire. Participants themselves participated by clicking on the link and their answers were sent directly to the secured servers of the faculty to which only the involved researchers had access. All participants were informed of the purpose of the study and that participation was voluntary. Consent for participation and processing the data could be derived by starting the survey. Participants could withdraw at any moment in the process. Data that had been collected up to that point were stored and used for analysis. This approach was chosen in order to prevent loss of this data and on the assumption that participants would have already stopped at earlier questions had they not wanted to answer them. The research was conducted in accordance with the principles expressed in the Declaration of Helsinki and the General Data Protection Regulation.

## Results

In total, 248 respondents started the survey, 184 from *subgroup agri* and 64 from *subgroup other.* After the questions about the selection criteria (professional pest controller for control of rodents), 151 respondents (105 *subgroup agri* and 45 *subgroup other*) remained. Of these respondents, 108 (73 *subgroup agri* and 35 *subgroup other*) completed the entire survey.

### Pest control

A majority of 62.1% (n = 154) of the respondents who started the survey (n = 248) indicated that their company contracts an external professional pest controller (see Table 1). The other respondents indicated that they had an in-company pest controller with a licence (n = 23; 9.3%) or that pest control was carried out a company employee without a professional control licence (n = 71; 28.6%). Of the companies where control was managed by an external professional, 116 (75.3%) had a permanent contract with the controller and 38 (24.7%) made use of the services on a flexible basis.

**Table 1.** Overview of the sectors where respondents (n = 248) are working in, whether companies contract an external professional pest controller, which animals are controlled, and whether the company has a protocol for pest control

	External professional controller		Animals controlled				Protocol		
Sector (n)	Yes	No	Insects	Rodents	Birds	Other	Yes	No	Don’t know
Agriculture (184)	105	79	58	183	14	5	74	90	20
Animal shelter (20)	13	7	2	18	1	1	8	10	2
Food processing (8)	8	0	4	8	2	0	4	4	0
Municipality (7)	3	4	3	4	1	1	4	2	1
Catering industry (7)	6	1	3	7	0	0	3	4	0
Zoos (5)	3	2	1	4	0	2	3	1	1
Bakery, butchery (3)	3	0	2	2	0	0	1	1	1
Hospitality (1)	1	0	0	1	0	0	0	1	0
Healthcare (1)	1	0	0	1	0	0	0	1	0
Various (12)	11	1	8	11	5	2	8	2	2
**Total (248)**	**154**	**94**	**81**	**239**	**23**	**11**	**105**	**116**	**27**

The four main reasons given for contracting an external pest controller were as follows: compliance with regulations (n = 89; 57.8%); prevention of economic losses or damage (n = 72; 46.8%); contribution to a nice and healthy environment (including animal health) (n = 68; 44.2%); and contribution to food safety (n = 63; 40.9%). Other reasons given were no in-company knowledge about pest control (n = 48; 31.2%), compliance with a quality label (n = 45; 29.2%) and contribution to nature conservation (n = 12; 7.8%). Some respondents (n = 12; 7.8%) gave other reasons not provided in the multiple-choice list provided. Reasons mentioned were that it was not permitted to carry out control using anticoagulant rodenticides one-self (due to IPM regulations), to avoid future costs, a lack of time, for fire safety and to prevent guests from seeing rats and mice. Among respondents from the agricultural sector, the most popular reason was prevention of economic losses/damage (n = 55; 52.4%), followed by compliance with regulations, food safety and a healthy environment.

The majority (n = 239; 96.4%) of the respondents had rodent management taking place in their company. Almost half of the respondents (n = 116; 46.8%) indicated they did not have a protocol for pest control. In 105 (42.3%) of the cases, the company had such a protocol and 27 (10.9%) respondents did not know. Almost half (n = 50) of the respondents with a protocol provided a short description of what it entailed. These typically contain information about preventive measures, such as cleaning, storing food and feed. Furthermore, protocols contained information regarding the frequencies of monitoring and control, log sheets, control routes within the company, communication agreements, lists with advice, control methods and agreements about responsibilities. Lastly, certain protocols were based on quality or sector labels and norms, such as the ‘Better Life Label’ of the Dutch Society for the Protection of Animals (Beter Leven Keurmerk), ‘IKB Nederland’ (a Dutch quality system for animal production), protocols for hygiene and food safety or IPM protocols.

### Demographics

The majority (75.9%) of respondents who completed the entire survey (n = 108) were male, especially in *subgroup agri* (see Table 2). Most respondents (75%) were aged between 41 and 67. There was an equal distribution of respondents with practical or theoretical education. Within the *subgroup agri*, practical education was selected the most (63%) and within *subgroup other* theoretical education (73.5%). Most respondents (86.1%), and especially respondents in the *subgroup agri* (90.4%) indicated they had one or more companion or hobby animals.

**Table 2.** Overview of respondent demographics. Data were obtained through an online survey among professional clients of Dutch pest controllers

	Subgroup agri n (%)	Subgroup other n (%)	Total n (%)
**Gender**			
Male	59 (80.8%)	23 (65.7%)	82 (75.9%)
Female	13 (17.8%)	12 (34.3%)	25 (23.2%)
Other	1 (1.4%)	0 (0%)	1 (0.9%)
**Age class**			
≤30 years	9 (12.3%)	2 (5.7%)	11 (10.2%)
31–40 years	9 (12.3%)	6 (17.1%)	15 (13.8%)
41–50 years	23 (31.5%)	10 (28.6%)	33 (30.6%)
≥51 years	32 (43.9%)	17 (48.6%)	49 (45.4%)
**Education**			
Practical education	46 (63.0%)	9 (26.5%)	55 (51.4%)
Theoretical education	27 (36.0%)	25 (73.5%)	52 (48.6%)
**Companion or hobby animal**			
Yes	66 (90.4%)	27 (77.1%)	93 (86.1%)
No	7 (9.6%)	8 (22.9%)	15 (13.9%)

### Effects of grouping variables

No statistically significant differences were found for the grouping variables: gender, age class, educational level and owner of companion or hobby animals for most of the test variables. For some, effects of gender were noted and these results will be shown in the following paragraphs.

### General attitude towards liminal rodents

There was a significant difference between the scores of the five statements regarding the general attitude towards liminal rodents (n = 149, df = 4: χ*_2_* = 191.626; *P* < 0.0000005*, moderate effect; *W* = 0.32). The pair-wise comparisons of the five statements showed that all statements differed significantly from each other. Respondents agreed most with statement C (Presence of rats and mice is always undesirable) and were somewhat neutral regarding statement A (Rats and mice belong to nature).

Most respondents disagreed with statements B (Rats and mice deliver benefits to nature), D (Rats and mice have interests) and E (In pest management, people should take the interests of rats and mice into account), with median scores of 3, 1.3 and 1.2, respectively (Supplementary Table S3). Respondents were neutral regarding statement A (Rats and mice belong to nature) with a median score of 5.5. Most respondents agreed with statement C (Presence of rats and mice is always undesirable), with a median score of 9.2.

**Figure 1.**
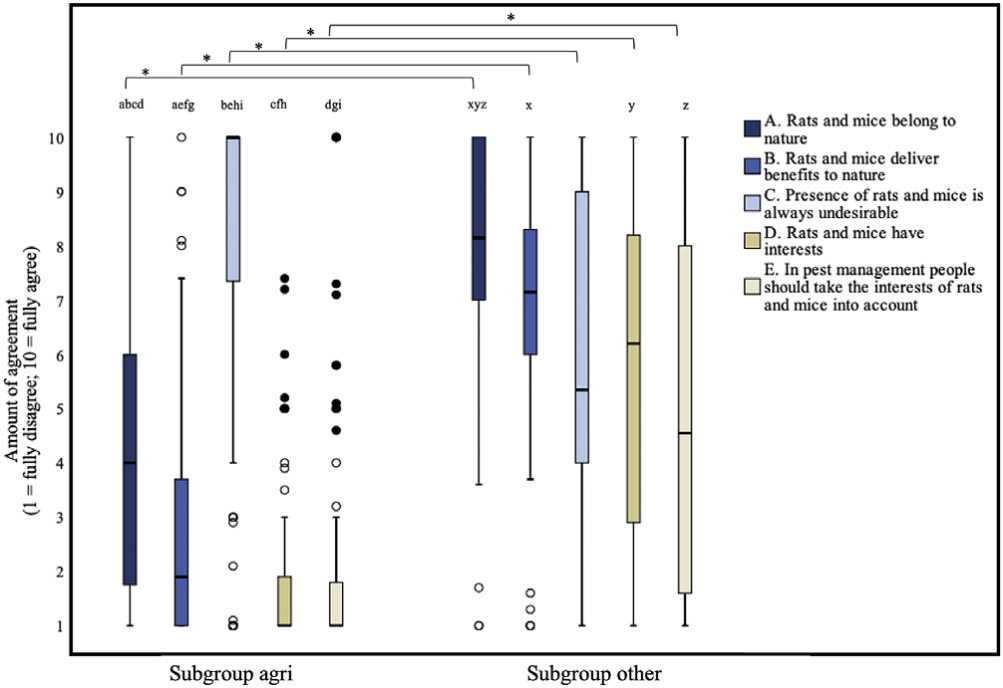
Box plots presenting the amount of agreement with statements A-E about the general attitudes towards rats (*Rattus rattus* and *Rattus norvegicus*) and mice (*Mus musculus*). The amount of agreement could be indicated on a 1 (fully disagree) to 10 (fully agree) continuous rating scale. The interests of rats and mice (statement D) were defined as living, freedom, and welfare. Data were obtained through an online survey among professional clients (n = 149) of Dutch pest controllers. Outliers and extreme cases are indicated with o and • respectively. Differences between subgroup agri (n = 103) and subgroup other (n = 46) that are statistically significant are indicated with an asterisk. Differences between two statements within subgroups that are statistically significant are indicated with letters.

Figure 1 shows boxplots with the responses of both the *subgroup agri* and the *subgroup other* to the five statements (A–E) about the general attitude towards rats and mice. For all statements there was a statistically significant difference between *subgroup agri* (n = 103) and *subgroup other* (n = 46) with *P*-values smaller than or equal to 0.000012* and moderate to large effects, ranging from |r| = 0.35 to |r| = 0.59 (see Supplementary Table S4). The *subgroup other* agreed more with statements A, B, D and E than *subgroup agri.* *Subgroup agri* agreed more with statement C. Women agreed more with statements D (median 3 vs. 1.4; *P* = 0.001088*, moderate effect, |r| = 0.31) and E (median 4.6 vs. 1.2; *P* = 0.000109*, moderate effect, |r| = 0.37) than men.

Within *subgroup agri*, there was a significant difference between the scores of the five statements regarding the general attitude towards liminal rodents (n = 103, df = 4: χ*_2_* = 216.911; *P* < 0.0000005*, large effect; *W* = 0.53). The pair-wise comparisons of the five statements showed that all statements differed significantly from each other (Supplementary Table S5), except for statement D vs. E.

The results found in *subgroup agri* are comparable with those found in the total group, with respondents disagreeing with all the statements, except for statement C (Presence of rats and mice is always undesirable).

Within *subgroup other*, there was also a significant difference between the scores of the five statements (n = 46, df = 4: χ*_2_* = 25.521; *P* = 0.000100*, small effect; *W* = 0.14). The pair-wise comparisons of the five statements showed that the scores for statement A vs. statement B, for statement A vs. statement D, and for statement A and E were significantly different from each other (Supplementary Table S6). Most respondents of *subgroup other* agreed with statements A and B, were somewhat neutral for statements C and E and tended to agree with statement D.

### Animal welfare

In the section about animal welfare, respondents were asked several general questions in relation to animal welfare (in rodent control). Most respondents (63.1% of the total) agreed that rats and mice were capable of experiencing pain (56% within *subgroup agri* and 79% within *subgroup other*), 12.3% of the total respondents (15.5% of the respondents within *subgroup agri* and 5.3% within *subgroup other*) did not agree with this and 24.6% (28.5% of the respondents within *subgroup agri* and 15.7% within *subgroup other*) did not know or had no opinion.

**Figure 2.**
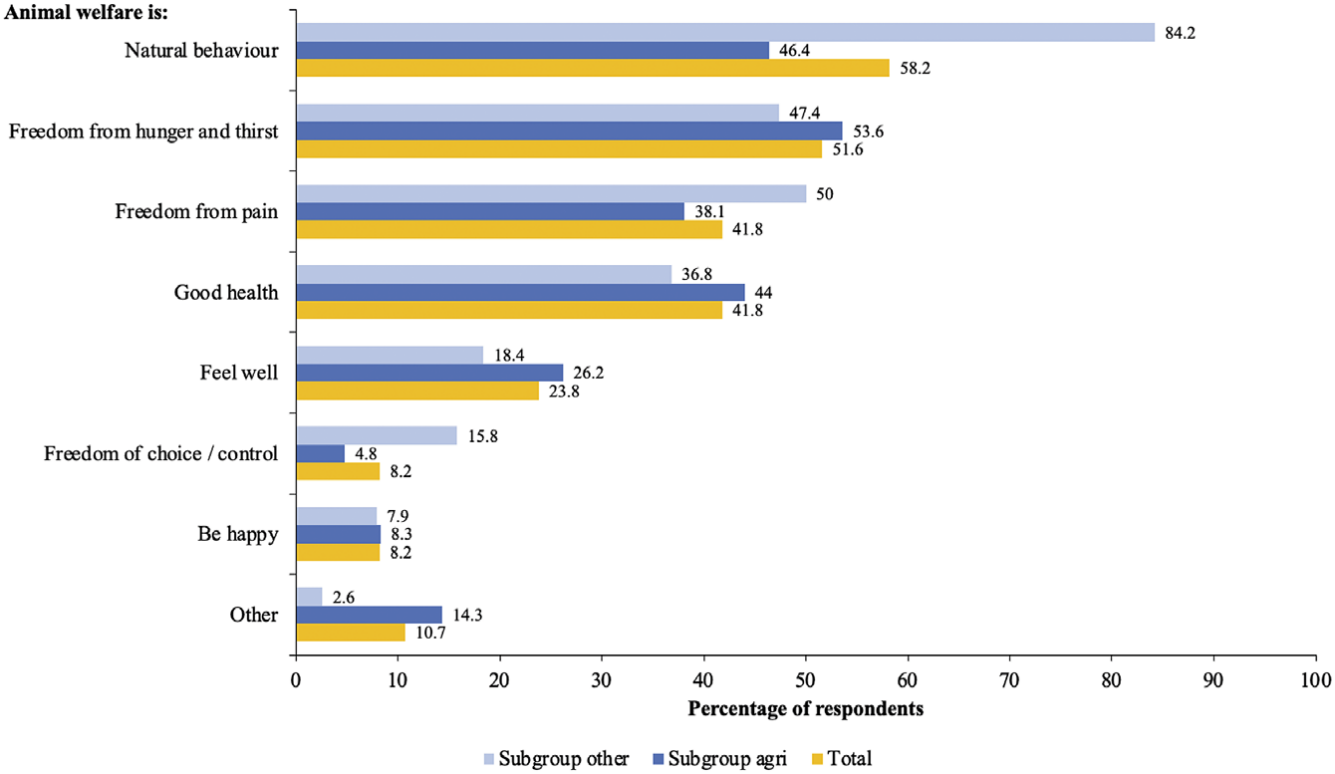
Bar chart presenting aspects being important for animal welfare according to clients of Dutch pest controllers. Respondents could choose a maximum of three aspects being important for their understanding of animal welfare. The numbers show the percentage of respondents who chose a certain aspect. A distinction is made between respondents within subgroup other (n = 38), within subgroup agri (n = 84) and within the total group of respondents (n = 122). In the survey, natural behaviour was mentioned as the ‘Possibility to show natural behaviour’.

Respondents were asked to select (maximum three) aspects they considered to be important for animal welfare according to them. The aspects provided in the list were mainly based on the Five Domains Model (Mellor *et al.* 2020) and were formulated in easily understandable terms. As shown in Figure 2, the aspect ‘Natural behaviour’ was mostly chosen by 58.2% of all respondents (n = 122), followed by ‘Freedom from hunger and thirst’ (51.6% of all respondents). Within the *subgroup other,* ‘Natural behaviour’ was chosen most by 84.2% of the respondents, followed by ‘Freedom from pain’ (50% of respondents) and ‘Freedom from hunger and thirst’ (47.4% of respondents). Within the *subgroup agri*, the aspect chosen most was ‘Freedom from hunger and thirst’ by 53.6% of the respondents, followed by ‘Natural behaviour’ (46.4% of respondents) and ‘Good health’ (44% of respondents).

Most respondents (n = 68; 55.7%) selected three answers. In total, 25.6% respondents (mostly from the *subgroup agri*) only selected one aspect. In this case the most popular aspect was ‘Other’with 9% of the respondents choosing it (mostly from *subgroup agri*), followed by ‘Natural behaviour’ and the other aspects that could be selected. In total, 10.7% of the respondents selected the aspect ‘Other.’ Additional texts provided with this answer suggested that these respondents felt that animal welfare was not of importance for rats and mice, or they provided another definition for animal welfare or didn’t know what to answer.

Respondents were asked to indicate how often they considered the welfare of pest rats and mice. Almost half of the respondents (47.5%) indicated that they never considered the welfare of rats and mice. Of the respondents, 23.8% seldomly did, 12.3% sometimes did, 12.3% often did and 4.1% always did. The majority of *subgroup agri* (58.3%) indicated that they never considered the welfare of pest rats and mice, whereas the majority (28.9%) of *subgroup other* indicated they considered it sometimes.

When asked to indicate what aspects are important in considering the welfare of pest rats and mice, most respondents in *subgroup other* (60.5%) chose ‘Preventive methods.’ In the *subgroup agri* this aspect was only chosen by 19% of the respondents. In this subgroup, the aspects chosen most were ‘Killing animals fast’ (36.9%) and ‘Killing animals fast and painless’ (33.3%). In *subgroup other* these two aspects were only chosen by 18.4% of the respondents for each one. ‘Killing animals painless’ was chosen by only 2.4% and the option ‘other’ by 5.8% of the respondents in *subgroup agri.* None of the total respondents chose ‘Catch and release.’

Most respondents (54.9%) indicated that they thought their company or organisation paid sufficient attention to the welfare of rats and mice (Table 3, statement F). Half (50%) of the respondents in the total group disagreed with the statement that pest controllers should pay attention to the welfare of rats and mice in their job (Table 3, statement G). The majority (59.5%) of respondents from *subgroup agri* disagreed with this statement, while the majority (60.6%) of the *subgroup other* agreed with it. The majority (63.1%) of the respondents in the total group and in both subgroups (63.1% each) agreed that the pest controller paid sufficient attention to the welfare of rats and mice (Table 3, statement H).

**Table 3.** Amount of agreement with three statements (F–H) about the importance of welfare of rats (*Rattus rattus* and *Rattus norvegicus*) and mice (*Mus musculus*) in pest control. The number of respondents that indicates the option chosen most frequently is displayed in **bold**

	Amount of agreement Number (%)				
Statement	Fully disagree	Disagree	Neutral	Agree	Fully agree
**F. My company pays sufficient attention to the welfare of rats and mice**					
Total (n = 122)	10	13	32	**42**	25
	(8.2%)	(10.7%)	(26.2%)	**(34.4%)**	(20.5%)
Subgroup agri (n = 84)	8	11	19	**26**	20
	(9.5%)	(13.1%)	(22.6%)	**(31%)**	(23.8%)
Subgroup other (n = 38)	2	2	13	**16**	5
	(5.3%)	(5.3%)	(34.2%)	**(42.1%)**	(13.1%)
**G. Pest controllers should pay attention to the welfare of rats and mice in their job**					
Total (n = 122)	**34**	27	27	24	10
	**(27.9%)**	(22.1%)	(22.1%)	(19.7%)	(8.2%)
Subgroup agri (n = 84)	**29**	21	23	9	2
	**(34.5%)**	(25%)	(27.4%)	(10.7%)	(2.4%)
Subgroup other (n = 38)	5	6	4	**15**	8
	(13.2%)	(15.8%)	(10.5%)	**(39.5%)**	(21.1%)
**H. The pest controller working for my company pays sufficient attention to the welfare of rats and mice**					
Total (n = 122)	6	9	30	**53**	24
	(4.9%)	(7.4%)	(24.6%)	**(43.4%)**	(19.7%)
Subgroup agri (n = 84)	4	5	22	**33**	20
	(4.8%)	(5.9%)	(26.2%)	**(39.3%)**	(23.8%)
Subgroup other (n = 38)	2	4	8	**20**	4
	(5.3%)	(10.5%)	(21.1%)	**(52.6%)**	(10.5%)

**Figure 3.**
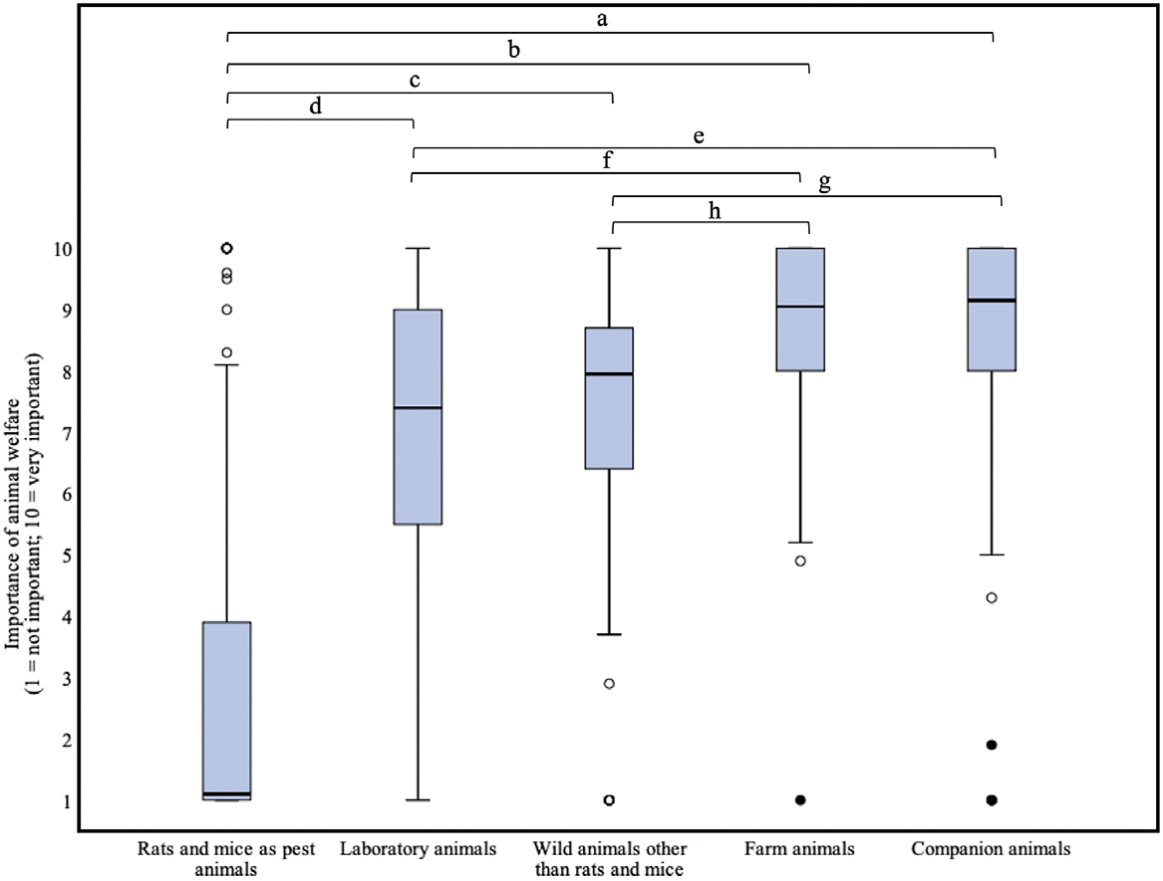
Box plots presenting the scored importance of animal welfare for five different categories of animals according to 122 professional clients of Dutch pest controllers participating in an online survey about the treatment of rats (*Rattus rattus* and *Rattus norvegicus*) and mice (*Mus musculus*). The importance could be indicated on a 1 (not important) to 10 (very important) continuous rating scale. Outliers and extreme cases are indicated with o and •, respectively. Statistically significant differences between two animal categories are indicated with letters.

Respondents scored the importance of animal welfare significantly differently depending on animal category/context (Figure 3) (n = 122, df = 4: χ^2^ = 282.103; *P* < 0.0000005*, large effect; *W* = 0.578). In the total group of respondents, the importance of animal welfare for rats and mice as pest animals, a median of 1.1, was scored significantly lower than for animals in other categories, with medians varying from 7.4 to 9.2 (*P* < 0.0000005*, large effects, |r| > 0.5).

Furthermore, significant differences were found between scores for animals in the other categories (see Figure 3, Supplementary Table S7). Women scored the importance of animal welfare for ‘Rats and mice as pest animals’ higher (median = 3.9; Q3–Q1 = 7.4–1.1) than men (median = 1; Q3–Q1 = 3–1) (n = 107; *P* = 0.001160*, moderate effect, |r| = 0.31). For the other categories, there were no differences between men and women.

Within the subgroups similar differences were found as in the total group of respondents (s*ubgroup agri*, n = 84, df = 4, χ^2^ = 223.890; *P* < 0.0000005*, large effect; *W* = 0.67; *subgroup other*, n = 38, df = 4, χ^2^ = 59.132; *P* < 0.0000005*, moderate effect; *W* = 0.39). Exact *P*-values and effect sizes for the pair-wise comparisons can be found in Supplementary Tables S8 and S9.

Respondents from the *subgroup agri* scored the importance of welfare for ‘Rats and mice as pest animals’ significantly lower (median of 1 compared to 5.5) than their counterparts from the *subgroup other* (*P* < 0.0000005*, large effect, |r| = 0.54). For the other animal categories, no significant differences were found between the subgroups.

### Welfare impact of control methods

Respondents (total group) saw differences in the estimated welfare impact of available control methods (n = 122, df = 9: χ*^2^* = 211.452; *P* < 0.0000005^*^, small effect; *W* = 0.19). According to the respondents, the methods ‘Glue board’ and ‘Trap and drown’ had a relatively high impact on welfare (medians of 8 and 7, respectively), whereas the other methods were deemed to have a relatively low or non-existant impact on welfare (medians ranging from 1 to 3). *Post hoc* testing (Supplementary Table S10) showed that the impact scores of ‘Glue board’ and ‘Trap and drown’ were rated significantly higher than all the other methods, except for ‘Trap and release.’ ‘Glue board’ and ‘Trap and drown’ do not differ significantly from each other. The impact of ‘EKO1000’ (Ekomille^®^, a trap in which rats fall into a solution of alcohol [participants were also provided with this description]; see Krijger *et al.* 2022) was scored significantly higher than ‘CO_2_ trap’ and ‘Shooting.’ Furthermore, the impact score of ‘Preventive methods’ was scored significantly lower than ‘Trap and release.’ No statistically significant differences were found between the other methods.

**Figure 4.**
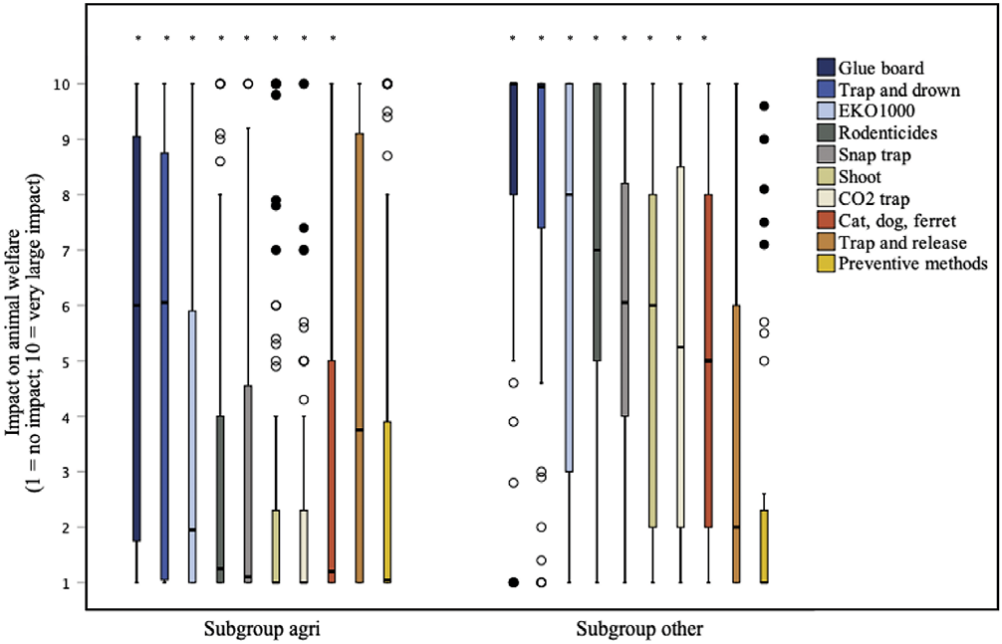
Box plots presenting the differences between subgroup agri (n = 84) and subgroup other (n = 38) in scored welfare impact of ten methods for the control of rats (*Rattus rattus* and *Rattus norvegicus*) and mice (*Mus musculus*), according to 122 clients of Dutch pest controllers. The impact could be indicated on a 1 (no impact) to 10 (very large impact) continuous rating scale. Outliers and extreme cases are indicated with o and •, respectively. Each method that differs significantly between subgroup agri and subgroup other is marked with an asterisk.

Respondents from the *subgroup agri* scored the impact of most methods lower than those from the *subgroup other* (Figure 4, Supplementary Table S11). No statistically significant differences between *subgroup agri* and *subgroup other* were found for ‘Trap and release’ and ‘Preventive methods.’

Respondents within the *subgroup agri* saw differences in the welfare impact of available control methods (n = 84, df = 9: χ*^2^* = 149.131; *P* < 0.0000005*, small effect; *W* = 0.20).

The impacts of ‘Glue board’ and ‘Trap and drown’ were not scored significantly differently from each other. The impact scores of these two were significantly higher than all the other methods, except for ‘Trap and release.’ The impact of ‘Trap and release’ was scored significantly higher than ‘CO_2_ trap.’ For all the pair-wise comparisons, see Supplementary Table S12.

Respondents within the *subgroup other* saw differences in the welfare impact of available control methods (n = 38, df = 9: χ^2^ = 104.413; *P* < 0.0000005^*^, moderate effect; *W* = 0.31). Within this subgroup, less differences were found though compared to the *subgroup agri.* For all pair-wise comparisons, see Supplementary Table S13.

### Weight of animal interests per situation

Besides the scoring of control methods in terms of their impact on animal welfare, respondents were also asked to indicate how important they considered animal interests (such as animal welfare) within the context of twelve different real-life scenarios related to rodent control. Respondents indicated that in different situations, a different weight should be attributed to animal interests (n = 109, df = 11: χ^2^ = 261.142; *P* < 0.0000005*, small effect; *W* = 0.22).

*Post hoc* testing (Supplementary Table S14) showed that whereas animal interests were deemed to be of almost no importance, for example, ‘Mice in a hospital kitchen’ (median of 1), animal interests mattered much more when dealing with ‘Mice in a private backyard’ (median of 5). For ‘Mice in a hospital kitchen’, the weight of animal interests was scored significantly lower compared to all other situations, except for ‘Mice in a supermarket’, ‘Mice on a pig farm’ and ‘Rats on a children’s farm’. Besides the differences between scenarios, the results indicated that respondents saw differences between rats and mice. For example, the weight of interests of ‘Mice in a private backyard’ was scored significantly higher than the interests of ‘Rats in a private backyard.’

**Figure 5.**
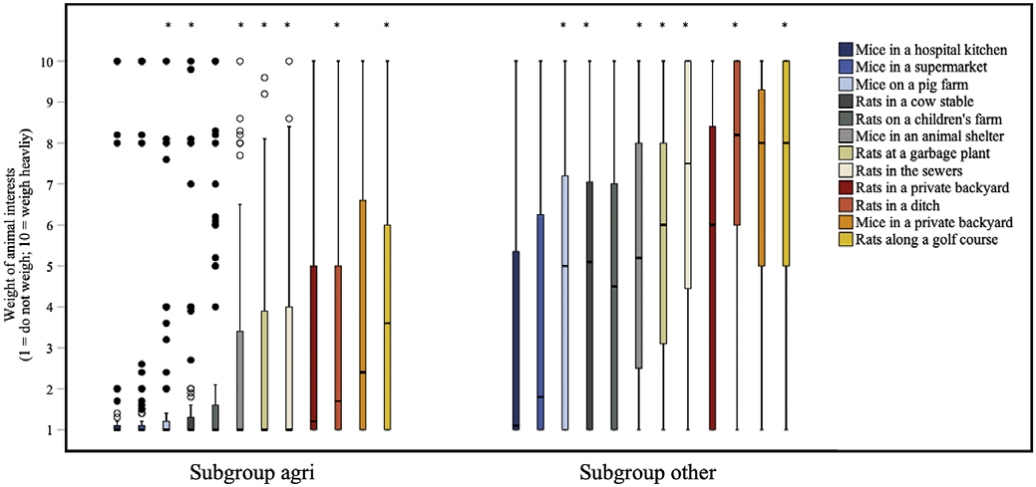
Box plots presenting the differences between subgroup agri (n = 74) and subgroup other (n = 35) in the weight of interests of rats (*Rattus rattus* and *Rattus norvegicus*) and mice (*Mus musculus*) in twelve different real-life scenarios, according to 109 clients of Dutch pest controllers. The interests of rats and mice were defined as living, freedom, and welfare. Weight of interests could be indicated on a 1 (do not weigh) to 10 (weigh heavily) continuous rating scale. Outliers and extreme cases are indicated with o and •, respectively. Each scenario that differs significantly between subgroup agri and subgroup other is marked with an asterisk.

Respondents from the *subgroup agri* gave a lower score than respondents from the *subgroup other* for most of the scenarios, with the exception of: ‘Mice in a hospital’, ‘Mice in a supermarket’, ‘Rats on a children’s farm’, ‘Rats in a private backyard’ and ‘Mice in a private backyard’ (Figure 5, Supplementary Table S15). For those scenarios the weight of animal interests was scored higher by *subgroup other* compared to *subgroup agri.*

Within the subgroups, similar differences as in the total group of respondents were found between the weight of animal interests in real-life scenarios (*subgroup agri*: n = 74; *P* < 0.0000005*, df = 11, χ^2^ = 168.001, small effect; *W* = 0.21; *subgroup other*: n = 35; *P* < 0.0000005*, df = 11, χ^2^ *=* 111.674, small effect; *W* = 0.29). In Supplementary Tables S16 and S17, the exact *P-*values and effect sizes can be found for the pair-wise comparisons per subgroup. Within *subgroup other*, the weight of animal interests for ‘Rats in a private backyard’ was scored significantly higher by women (median score of 8.7) compared to men (median score of 4.3) (*P* = 0.000591*, large effect, |r| = 0.56).

Of all respondents, 44% (40.5% within *subgroup agri* and 51.4% within *subgroup other*) did not face problems when weighing animal and human interests in practice. Of all respondents 39.4% (and 37.8% within *subgroup agr*i and 42.9% in *subgroup other*) did face problems and 16.5% (21.6% within *subgroup agri* and 5.7% within *subgroup other*) did not know. Respondents could provide a brief description of the problems faced as open text. Problems indicated are related to the following aspects: the location of the company or design of the public spaces being a challenge to the prevention or control of rodents; the timing of control (it should be as early as possible to prevent further suffering of rodents); preventive methods not being sufficient, being too complicated, too expensive or challenging to involve personnel in prevention; the presence of strict regulations for food safety and human or animal health (zero tolerance areas); too strict regulations or disagreement with IPM regulations; unacceptable nuisance when rodent welfare is taken into account; human interests being more important than rodent interests; expressions of negative attitudes towards rodents (e.g. they simply need to be killed and their welfare does not matter).

## Discussion

This research was performed with the aim of gaining an insight in the attitudes of clients of Dutch pest controllers as regards the management of commensal rodents and their subsequent welfare. The research served also to verify the findings of an earlier survey study among pest controllers (Van Gerwen *et al.* 2020) and explore whether the ideas of the pest controllers match those of their clients. The study data may be of use in four ways. Firstly, through providing information for the further development and implementation of preventive measures in rodent management practice. Secondly, as findings provide input for the development of an assessment framework to support ethical decision-making in the control of liminal rodents. Thirdly, the findings may be helpful in improving the communication and co-operation between professional pest controllers and their clients. And, finally, the study offers an additional insight into the field of human attitudes towards non-human animals and how these relate to profession and gender and the specific animals in question.

### Attitudes and opinions about animal welfare

The general attitude towards rats and mice in the total group of respondents and especially in the *subgroup agri* was relatively negative, also in comparison to the attitudes of the pest controllers from the previous study (Van Gerwen *et al.* 2020). In the *subgroup agri* most respondents disagreed with the statements that rats and mice were part of nature, that they delivered benefits to nature, that they had interests, and that people should consider the interests of rats and mice in rodent management. The latter two statements form a crucial aspect of an assessment framework in which animal and human interests are under consideration since the assumption that rats and mice do not have interests worth considering renders the weighing of interests seemingly irrelevant. Furthermore, 15.5% of the respondents in this subgroup (compared to 5.3% in the *subgroup other*) did not think that rats and mice were capable of experiencing pain. Another 28.5% in *subgroup agri* and 15.7% in *subgroup other* indicated that they did not know whether rats and mice experienced pain. This equates to 44% of *subgroup agri* and 21% of *subgroup other* ignoring the fact that rats and mice are sentient beings (Duncan 2006; Proctor 2012), a concept that has far-reaching consequences for the moral status of non-human animals and any consideration of their interests (e.g. welfare) (Proctor 2012; DeGrazia 2020). Furthermore, freedom from pain was not included in top three of aspects of importance for animal welfare in the *subgroup agri* in which respondents found freedom from hunger and thirst the most important aspect, followed by natural behaviour.

In our previous study (Van Gerwen *et al.* 2020) we found that Dutch pest controllers felt that rodent welfare mattered, although less so than the welfare of animals in other contexts, and they indicated that they considered rodent welfare in their job. The respondents from *subgroup other* in the current study gave answers more broadly comparable to the pest controllers from the previous study than the respondents from *subgroup agri.* Overall, this latter subgroup indicated they found the interests of pest rodents unimportant, and the majority never considered the welfare of rats and mice, while in *subgroup other* the majority did consider it and deemed it to be important, although less so than the welfare of animals in other contexts. In the *subgroup other* there was greater variation in the answers provided, especially for the statement that people should take the interests of rats and mice into account. This variation is likely due to the variation in sectors represented in this subgroup.

Respondents from the *subgroup agri* disagreed with the statement that pest controllers should pay attention to rodent welfare, while respondents from the *subgroup other* agreed it. Both groups were of the opinion the company and the pest controller both paid sufficient attention to rodent welfare. This is remarkable since the subgroups thought differently about the importance of animal welfare. It may suggest that the respondents were satisfied with the pest controller they had, and that the pest controller acted in accordance with the views of his/her clients.

For a number of survey questions, men and women answered differently. Female respondents placed greater importance than men on the overall interests and welfare of rats and mice.

The general attitudes and the importance of welfare found here are in line with the findings from previous studies. Firstly, men and women also had differing opinions in other studies (Herzog 2007; Taylor & Signal 2009; Herzog *et al.* 2015; Bradley *et al*. 2020), with women generally showing more concern for animal welfare and protection compared to men. However, Taylor and Signal (2009) found that gender did not influence attitudes towards pest animals. Here, we found that women did not respond differently to men in the majority of questions, though. Our results may be due to an overrepresentation of men in our study, especially in the *subgroup agri.* The differences found between men and women may be more of a subgroup effect than a gender effect, since no effects of gender were found within the subgroups.

Further research into the attitudes of men and women towards pest animals in particular is recommended, ideally with an equal distribution of men and women. Also, different questions regarding rodent control should be combined with and compared to scores on animal attitude scales (Animal Attitude Scale, Animal Purpose Questionnaire or Pet Pest Profit Scales) and/or the Speciesism Scale.

Secondly, differences based on occupation or profession were also found by Taylor and Signal (2005) whereby people with an agriculturally related profession (e.g. livestock farmer) were found to score lower on the Animal Attitude Scale. However, in another study (in 2009), the same authors found that employment within a primary industry, such as the agricultural sector, only affected the Profit subscale, not the Pet and Pest subscales. In that study profession was not seen to affect attitudes towards pest animals. The differences found may be a result of having a very large group of respondents from the agricultural sector compared to the group of other respondents which containing an array of differing professions. For future research it would be interesting to compare more professions than simply two groups (agricultural vs. non-agricultural) and use equal numbers per profession group.

Thirdly, we also found an effect of animal category on attitudes. In both subgroups the importance of welfare was scored lower for rats and mice compared to other animal categories (e.g. pet animals, lab animals and farm animals). Previous studies (Taylor & Signal 2009; Herzog 2011; Bradley *et al*. 2020) have also found these differences in attitudes towards animals depending on context and species indicating that pest animals are generally considered to be of less value than animals in other contexts.

Attitudes of people towards animals (in different contexts) and the assessment of these attitudes are complex and incorporate different factors and methods (Mankad *et al.* 2019). Furthermore, nationality and cultural background may influence views and opinions on animal welfare (Phillips *et al.* 2012; Tomasevic *et al*. 2020; Randler *et al.* 2021). In this study we only asked Dutch people about their attitudes via different statements. We did not ask them about factors such as their underlying beliefs, feelings or moral intuitions such as disgust (Haidt 2001) since this lay beyond the scope of our study. To gain a more complete assessment and understanding of such attitudes it would be useful to look closer into the different aspects of attitudes and, for example, scrutinise the knowledge and experiences that people have regarding this topic.

### Welfare impacts of methods and decisions in practice

The welfare impacts of different control methods were scored relatively low by the total group of respondents, especially when compared to the scores of pest controllers from the previous study (Van Gerwen *et al.* 2020). Only the welfare impacts of the methods glue trap and trap and drown were scored fairly high. The pest controllers from the previous study scored more in line with recent scientific findings (Baker *et al.* 2022; Krijger *et al.* 2022; De Ruyver *et al.* 2023). It should be noted however, that respondents from *subgroup other* also scored more in line with these scientific findings. Respondents from *subgroup agri* generally scored the welfare impact of control methods in lower than respondents from *subgroup other* and pest controllers from the previous study. Furthermore, respondents from the *subgroup agri* indicated for 7 out of 10 methods (including rodenticides and the EKO1000 trap [Ekomille^®^, a trap in which rats fall into a solution of alcohol; participants were also provided with this description]), that these methods do not impact upon welfare. The exact mode of death of the EKO1000 trap, however, was not known while the survey was being undertaken (early 2020). In the study by Krijger *et al*. (2022) rats were found to drown, and the welfare impact score is estimated to be severe or extreme. The respondents from *subgroup agri* were likely of the opinion that the EKO1000 trap and its use of an alcohol solution were better for animal welfare, compared to ‘trap and drown.’ Respondents from the *subgroup other* did not make a distinction between the impact of these two methods. Rodenticides were also scored no (or a very low) impact by respondents from *subgroup agri.* This contrasts with the high impact rodenticides were assigned in various studies (Broom 1999; Mason & Littin 2003; Baker *et al.* 2022; De Ruyver *et al.* 2023), typically as a direct result of their slow mode of death (several days). Since respondents from *subgroup agri* considered the fast and painless killing of rodents to be important aspects for the welfare of rats and mice, it can be questioned whether they were aware of the mode of action and animal welfare consequences of rodenticides. Furthermore, the scores from respondents in the *subgroup agri* may be a reflection of the respondents’ attitudes towards rats and mice and their opinions on the importance of rodent welfare. Respondents from *subgroup other* considered preventive methods the most important aspect for welfare of rats and mice in rodent control. This may be one of the reasons why they scored the welfare impact of lethal methods higher than respondents from the *subgroup agri.* The method ‘catch and release’ was seen as one with a degree of impact by respondents from the *subgroup agri.* In *subgroup other* this method received a much lower impact. The catch and release method has not been evaluated scientifically. Despite this seeming to be a far more animal-friendly method since it does not kill rodents, it may not be so in practice. It is unclear what chances of survival the caught animals have, and the inspection frequency of the cage trap greatly influences the welfare (De Ruyver *et al.* 2023).

In our study we did not ask respondents for the reasons behind their scorings and their level of knowledge regarding the methods in question. This may be useful for further research though, since it could also contribute to a better education of people in the light of IPM and prevention. The educational aspect was also mentioned by Dutch pest controllers (Van Gerwen *et al.* 2020) as an important aspect for a better implementation of preventive methods.

Respondents indicated that the importance of rodent interests (e.g. welfare) was dependent upon the situation. The weight attributed to welfare may say something about the tolerance level towards the presence of rats and mice in different real-life situations. Respondents from the *subgroup agri* scored the importance of animal interests lower than those from the *subgroup other* for most of the situations. Interestingly, scores between subgroups did not differ significantly for situations in which human health risks typically occur and where a zero tolerance could be desirable, e.g. in the hospital kitchen, in the supermarket and at a petting zoo (children’s farm).

Respondents from the *subgroup agri* seemed to consider rodent interests to be of generally low importance, while respondents from the *subgroup other* found the interests of rats and mice important for at least some of the situations described in the survey. This is comparable to the views of the pest controllers in our previous study (Van Gerwen *et al.* 2020). The scoring in *subgroup agri* may be, as with the scoring of welfare impact of control methods, a reflection of their views on rats and mice. Most respondents in this subgroup did not agree in the first place with the statement that rats and mice have interests (e.g. welfare) and did not consider rodent welfare important. If one does not assume that animals have interests, one is also likely to say that interests do not matter.

Whether or not the presence of rodents is acceptable, and the formulation of tolerance levels forms an important first step in the decision-making process for a more ethical rodent management (Yeates 2009) and to safeguard animal welfare in rodent control. From the present study it seems that the location at least has some effect on the tolerance level, especially in the *subgroup other.* More factors (e.g. general attitude towards animals and rodents, views on animal welfare, gender, occupation, pet ownership, species, and numbers of rodents present) may also however have an effect and further research is needed to investigate what factors contribute to the definition of threshold levels for rodent presence or socio-cultural carrying capacity for rats and mice (Van Gerwen *et al*. 2021).

### Limitations of the study

Firstly it should be noted that the respondents of this survey do not represent the Dutch population of pest controller clients (this was also not aimed for). Results should therefore be used in an indicative way without generalising conclusions. Further research is needed to draw more general conclusions.

There was an overrepresentation of respondents from the agricultural sector. On the one hand this may be due to a better distribution of the survey link within the agricultural sector. On the other, it may be due to a high degree of involvement in the debate about rodent management and pest control in a broader sense and the changes in IPM regulations in The Netherlands. In The Netherlands, IPM certification is a prerequisite for the use of anticoagulant rodenticides since 2017 (the regulation also includes the use of cholecalciferol) to control rats outside buildings. From 2023 onwards, this will also be the case for the control of mice and rats both inside and outside buildings. This means that private persons are no longer allowed to use these types of rodenticides for controlling rats or mice themselves. Only licensed professionals can still use do so. Farmers are allowed to use rodenticides in the future on their own farm, but only if they are IPM-certified and have followed a specific course for this certification.

There was an underrepresentation of respondents from several other professional sectors, among which hospitality and health care services (only one respondent per group), supermarkets and garbage processing (0 respondents from these groups). This may be a result of limited distribution of the survey within these sectors or by a limited level of involvement with or interest in the topic, which is a signal that professional pest controllers also give (Van Gerwen *et al.* 2020). It may also have been due to the onset of the COVID-19 pandemic at the beginning of 2020. During this period, people in the hospitality and health sectors were especially occupied with COVID-related activities and concerns. For further research we would recommended distributing similar surveys wider and within a more diverse group of sectors.

In this study different grouping variables were used for analysis. For most of the grouping variables no effects were found. This may be due to low or unequal numbers of respondents within different variable categories (e.g. women, respondents under 40 years and respondents without pets). Some differences in attitudes were found between genders and these have been discussed previously.

A total of 248 respondents started our survey, but only 151 remained for participation after the selection criteria (making use of a professional pest controller for control of rodents). We did not ask why companies without a professional controller chose this approach and whether they had a certain level of knowledge themselves. This information would be valuable to have though, since a degree of knowledge and experience is required to adequately perform rodent management and ensure a responsible use of (lethal) control methods with varying impacts on welfare (Baker *et al.* 2022; De Ruyver *et al.* 2023).

For respondents from the agricultural sector no information was available regarding the type of farm (e.g. animal species, type of crops, open or closed barn) or type of company in the agricultural supply chain. This information was not requested in the survey but could be relevant for further research since open barns with animal feed present are more challenging locations for pest control and prevention than closed barns. This may influence the answers that respondents provide.

### Animal welfare implications

The current study is part of a larger study that also looked at client attitudes towards IPM and the application of preventive measures. The complete study serves to: (1) verify the findings of the previous study among pest controllers; and (2) explore whether the ideas of the pest controllers match client views. Furthermore, (3) the outcomes will be used to develop an assessment framework that can support ethical decision-making in the practice of rodent control and be used both by pest controller and client. We consider the input of pest controllers and clients to be indispensable for the development and practical implementation of such a framework. Insight into opinions and attitudes of both pest controller and clients may contribute in this way to a more ethical rodent management where the moral position of rodents and rodent welfare are considered.

## Conclusion

The aim of the current study was to gain insight into the opinions and attitudes of clients of professional pest controllers (e.g. organisations and companies that engage a pest control contractor), regarding liminal rodents, rodent control, and rodent welfare. Survey respondents, especially within the agricultural sector, had a negative attitude towards commensal rats and mice. Respondents in the agricultural subgroup did not consider the welfare of these rodents important. They thought that the welfare impact of commonly used control methods was low, and had low tolerance levels towards rats and mice. This compared to the respondents from the *subgroup other,* who had a much more positive attitude towards rats and mice, considered rodent welfare more important, scored the welfare impact of methods higher and had greater tolerance levels. The respondents from the latter subgroup had a similar attitude compared to the pest controllers participating in a previous survey study. Although the findings of this study are not representative of Dutch pest controller clients on the whole, they provide useful information for the further development and, in particular, the practical implementation of preventive methods, a more animal-friendly rodent control and the development of an assessment framework to support ethical decision-making in the control of liminal rodents. Furthermore, the results can be helpful for the communication and co-operation between professional pest controllers and clients.

## Supplementary material

For supplementary material accompanying this paper visit https://doi.org/10.1017/awf.2023.35. click here to view supplementary material
