# Supplementary material for: Attitudes of clients of Dutch pest controllers towards animal welfare in the management of liminal rodents
Source: Anim Welf. 2023 May 26;32:e41. doi: 10.1017/awf.2023.35 (PMC10936359; doi:10.1017/awf.2023.35)
Supplement: Supplementary file 1 [file S0962728623000350sup001.pdf]

Table S1 Overview of the corrected thresholds for statistical significance

Topic<br>● *number of test variables*	*Omnibus* tests<br>Friedman repeated measures test *or* Kruskall-Wallis test	*Post hoc* tests<br>Wilcoxon matched-pairs signed ranks test	Wilcoxon-Mann-Whitney test<br>*Omnibus* tests<br>Subgroup (Agri/Other) *or* Companion animal (Yes/No) *or* Gender (male/female) *or* Education (low/high)	Wilcoxon-Mann-Whitney test<br>*Post hoc* tests<br>Age group (4)
	$\gamma = t$ x o[1]	$\gamma = t$ x o x c	$\gamma = t$ x o	$\gamma = t$ x o x c
General attitude of liminal rodents				
● *5 test variables*				
γ	5 x 6 = 30	5 x 6 x 10 = 300	5 x 6 = 30	5 x 6 x 6 = 180
α_{adj}[2]	0.001708	0.000171	0.001708	0.000285
Animal welfare in context				
● *5 test variables*				
γ	5 x 6 = 30	5 x 6 x 10 = 300	5 x 6 = 30	5 x 6 x 6 = 180
α_{adj}	0.001708	0.000171	0.001708	0.000285
Welfare impact of methods				
● *10 test variables*				
γ	10 x 6 = 60	10 x 6 x 45 = 2700	10 x 6 = 60	10 x 6 x 6 = 360
α_{adj}	0.000855	0.000019	0.000855	0.000142
Weight of animal interest in different real-life scenarios				
● *12 test variables*				
γ	12 x 6 = 72	12 x 6 x 66 = 4752	12 x 6 = 72	12 x 6 x 6 = 432
α_{adj}	0.000071	0.000011	0.000071	0.000119

1 γ is the number of hypotheses tested (*omnibus* tests: 'number of test variables' (t) multiplied by 'the number of *omnibus* tests performed' (o); *post hoc* tests: 'number of test variables' (t) multiplied by 'the number of *omnibus* tests performed'(o) multiplied by 'the number of pair wise comparisons'(c) per test variable)

2 The formula for calculating the *adjusted alpha* is: $\alpha_{adj} = 1 - [1 - \alpha]^{1/\gamma}$, where $\alpha = 0.05$

Table S2 Overview of the corrected thresholds for statistical significance in the case of the subgroups *agri* and *other*

Topic<br>● *number of test variables*	*Omnibus* tests<br>Friedman repeated measures test *or* Kruskall-Wallis test	*Post hoc* tests<br>Wilcoxon matched-pairs signed ranks test	Wilcoxon-Mann-Whitney test<br>*Omnibus* tests<br>Companion animal (Yes/No) *or* Gender (male/female) *or* Education (low/high)	Wilcoxon-Mann-Whitney test<br>*Post hoc* tests<br>Age group (4)
	$\gamma = t$ x o[1]	$\gamma = t$ x o x c	$\gamma = t$ x o	$\gamma = t$ x o x c
General attitude of liminal rodents<br>● *5 test variables*				
γ	5 x 5 = 25	5 x 5 x 10 = 250	5 x 5 = 25	5 x 5 x 6 = 150
α_{adj}[2]	0.0002050	0.000205	0.002050	0.000342
Animal welfare in context<br>● *5 test variables*				
γ	5 x 5 = 25	5 x 5 x 10 = 250	5 x 5 = 25	5 x 5 x 6 = 150
α_{adj}	0.002050	0.000205	0.002050	0.000342
Welfare impact of methods<br>● *10 test variables*				
γ	10 x 5 = 50	10 x 5 x 45 = 2250	10 x 5 = 50	10 x 5 x 6 = 300
α_{adj}	0.001025	0.000023	0.001025	0.000171
Weight of animal interest in different real-life scenarios<br>● *12 test variables*				
γ	12 x 5 = 60	12 x 5 x 66 = 3960	12 x 5 = 60	12 x 5 x 6 = 360
α_{adj}	0.000855	0.000013	0.000855	0.000142

1 γ is the number of hypotheses tested (*omnibus* tests: 'number of test variables' (t) multiplied by 'the number of *omnibus* tests performed' (o); *post hoc* tests: 'number of test variables' (t) multiplied by 'the number of *omnibus* tests performed'(o) multiplied by 'the number of pair wise comparisons'(c) per test variable)

2 The formula for calculating the *adjusted alpha* is: α_{adj} = 1- $[1 - \alpha]^{1/\gamma}$, where $\alpha = 0.05$

Table S3 Exact P-values and effect sizes for general attitudes about liminal rodents for the total group of respondents.

	**A. RM belong to nature (5.5; 8.1-2.7)**	**B. RM deliver benefits to nature (3; 6-1)**	**C. Presence of RM is always undesirable (9.2;10-5)**	**D. RM have interests (1.3;4.1-1)**	**E. In pest management people should take the interests of RM into account (1.2;4-1)**
**A. RM belong to nature (5.5; 8.1-2.7)**		0.4472	0.2552	0.4853	0.4786
**B. RM deliver benefits to nature (3; 6-1)**	<0.0000005*		0.4014	0.3166	0.2886
**C. Presence of RM is always undesirable (9.2;10-5)**	0.000007*	<0.0000005*		0.4767	0.4784
**D. RM have interests (1.3;4.1-1)**	<0.0000005*	<0.0000005*	<0.0000005*		0.0897
**E. In pest management people should take the interests of RM into account (1.2;4-1)**	<0.0000005*	0.0000002*	<0.0000005*	0.122217	

Table S3: Exact (2-tailed) P-values (under de diagonal line) and absolute effect sizes (above the diagonal line) for differences between the amount of agreement with statements A-E about the general attitudes of rats (*Rattus rattus* and *Rattus norvegicus*) and mice (*Mus musculus*). The amount of agreement could be indicated on a 1 (fully disagree) to 10 (fully agree) continuous rating scale. Interests of rats and mice (statement D) were defined as living, freedom, and welfare. Data were obtained through an online survey among professional clients (n = 149) of Dutch pest controllers. Exact (2-tailed) P values were calculated for each pair of statements with the Wilcoxon matched-pairs signed ranks test for 2 dependent variables. Numbers in parentheses display the median and the interquartile range (Q3-Q1). Significant P-values are marked with an asterisk (*). Effect sizes (r) were calculated using the formula $r = z / \sqrt{n}$, where 'n' is the number of observations, in this case 2 x 149 = 298. Thresholds used for qualitative descriptions of effect size were: zero or nearly zero effect, $0 < |r| \leq 0.1$; small, $0.1 < |r| \leq 0.3$; moderate, $0.3 < |r| \leq 0.5$; large $0.5 < |r| \leq 0.7$; and very large, $|r| > 0.7$.

Table S4 Medians, Inter Quartile Ranges (Q3-Q1), exact P-values and effect sizes for the difference in general attitudes about liminal rodents between *subgroup agri* and *subgroup other*

	Subgroup agri Median (Q3-Q1)	Subgroup other Median (Q3-Q1)	Exact *p* value (2-sided)	Effect size |r|
**A. RM belong to nature**	4 (6-1.8)	8.2 (10-7)	0.101581	0.1600
**B. RM deliver benefits to nature**	1.9 (3.7-1)	7.2 (8.3-6)	0.004188	0.2739
**C. Presence of RM is always undesirable**	10 (10-7.4)	5.4 (9-4)	0.533642	0.0607
**D. RM have interests**	1 (1.9-1)	6.2 (8.2-2.9)	0.001088*	0.3108
**E. In pest management people should take the interests of RM into account**	1 (1.8-1)	4.6 (8-1.6)	0.000109*	0.3647

Table S4: Median, Inter Quartile Ranges (IQR, Q3-Q1), exact (2-tailed) P values (under de diagonal line) and absolute effect sizes (above the diagonal line) for differences in the amount of agreement with statements A-E about the general attitudes of rats (*Rattus rattus and Rattus norvegicus*) and mice (Mus musculus) between subgroup agri (n = 103) and subgroup other (n = 46). The amount of agreement could be indicated on a 1 (fully disagree) to 10 (fully agree) continuous rating scale. Interests of rats and mice (statement D) were defined as living, freedom, and welfare. Data were obtained through an online survey among professional clients (n = 149) of Dutch pest controllers. Exact (2-tailed) P-values were calculated for each statement with the Wilcoxon–Mann–Whitney test for independent variables. Significant P-values are marked with an asterisk (*). Effect sizes (r) were calculated using the formula $r = z / \sqrt{n}$, where 'n' is the number of observations, in this case 149. Thresholds used for qualitative descriptions of effect size were: zero or nearly zero effect, $0 < |r| \leq 0.1$; small, $0.1 < |r| \leq 0.3$; moderate, $0.3 < |r| \leq 0.5$; large $0.5 < |r| \leq 0.7$; and very large, $|r| > 0.7$.

Table S5 Exact P-values and effect sizes for general attitudes about liminal rodents for *subgroup agri*

	**A. RM belong to nature**	**B. RM deliver benefits to nature**	**C. Presence of RM is always undesirable**	**D. RM have interests**	**E. In pest management people should take the interests of RM into account**
**A. RM belong to nature**		0.4725	0.4216	0.5129	0.4902
**B. RM deliver benefits to nature**	<0.0000005*		0.5370	0.3598	0.2672
**C. Presence of RM is always undesirable**	<0.0000005*	<0.0000005*		0.5687	0.5543
**D. RM have interests**	<0.0000005*	<0.0000005*	<0.0000005*		0.0079
**E. In pest management people should take the interests of RM into account**	<0.0000005*	0.000076*	<0.0000005*	0.91313	

Table S5: Exact (2-tailed) P-values (under de diagonal line) and absolute effect sizes (above the diagonal line) for differences between the amount of agreement with statements A-E about the general attitudes of rats *(Rattus rattus and Rattus norvegicus*) and mice (*Mus musculus*). The amount of agreement could be indicated on a 1 (fully disagree) to 10 (fully agree) continuous rating scale. Interests of rats and mice (statement D) were defined as living, freedom, and welfare. Data were obtained through an online survey among professional clients (n = 149) of Dutch pest controllers. In this table the results of *subgroup agri* (n = 103) are displayed. Exact (2-tailed) P-values were calculated for each pair of statements with the Wilcoxon matched-pairs signed ranks test for 2 dependent variables. Significant P-values are marked with an asterisk (*). Effect sizes (r) were calculated using the formula $r = z / \sqrt{n}$, where 'n' is the number of observations, in this case 2 x 103 = 206. Thresholds used for qualitative descriptions of effect size were: zero or nearly zero effect, $0 < |r| \leq 0.1$; small, $0.1 < |r| \leq 0.3$; moderate, $0.3 < |r| \leq 0.5$; large $0.5 < |r| \leq 0.7$; and very large, $|r| > 0.7$.

Table S6 Exact P-values and effect sizes for general attitudes about liminal rodents for *subgroup other*

	**A. RM belong to nature**	**B. RM deliver benefits to nature**	**C. Presence of RM is always undesirable**	**D. RM have interests**	**E. In pest management people should take the interests of RM into account**
**A. RM belong to nature**		0.3815	0.3143	0.4182	0.4524
**B. RM deliver benefits to nature**	0.000087*		0.1781	0.2545	0.3443
**C. Presence of RM is always undesirable**	0.002022	0.088371		0.0217	0.1354
**D. RM have interests**	0.000011*	0.013374	0.839258		0.2428
**E. In pest management people should take the interests of RM into account**	0.000002*	0.000607	0.197554	0.018314	

Table S6: Exact (2-tailed) P-values (under de diagonal line) and absolute effect sizes (above the diagonal line) for differences between the amount of agreement with statements A-E about the general attitudes of rats (*Rattus rattus and Rattus norvegicus*) and mice (*Mus musculus*). The amount of agreement could be indicated on a 1 (fully disagree) to 10 (fully agree) continuous rating scale. Interests of rats and mice (statement D) were defined as living, freedom, and welfare. Data were obtained through an online survey among professional clients (n = 149) of Dutch pest controllers. In this table the results of *subgroup other* (n = 46) are displayed. Exact (2-tailed) P-values were calculated for each pair of statements with the Wilcoxon matched-pairs signed ranks test for 2 dependent variables. Significant P-values are marked with an asterisk (*). Effect sizes (r) were calculated using the formula $r = z / \sqrt{n}$, where 'n' is the number of observations, in this case 2 x 46 = 92. Thresholds used for qualitative descriptions of effect size were: zero or nearly zero effect, $0 < |r| \leq 0.1$; small, $0.1 < |r| \leq 0.3$; moderate, $0.3 < |r| \leq 0.5$; large $0.5 < |r| \leq 0.7$; and very large, $|r| > 0.7$.

Table S7 Exact P-values and effect sizes for importance of animal welfare between animal categories in the total group of respondents

	Rats and mice as pests (1.1;3.9-1)	Laboratory animals (7.4;9-5.5)	Other wild animals (8;8.7-6.4)	Farm animals (9.1;10-8)	Companion animals (9.2;10-8)
**Rats and mice as pests (1.1;3.9-1)**		0.5441	0.5547	0.5834	0.5770
**Laboratory animals (7.4;9-5.5)**	<0.0000005*		0.0664	0.4444	0.4373
**Other wild animals (8;8.7-6.4)**	<0.0000005*	0.3064		0.4742	0.4408
**Farm animals (9.1;10-8)**	<0.0000005*	<0.0000005*	<0.0000005*		0.0348
**Companion animals (9.2;10-8)**	<0.0000005*	<0.0000005*	<0.0000005*	0.6008	

Table S7: Exact (2-tailed) P-values (under de diagonal line) and absolute effect sizes (above the diagonal line) for differences in the importance of animal welfare for five different animal categories. Exact (2-tailed) P-values were calculated for each pair of methods with the Wilcoxon matched-pairs signed ranks test for 2 dependent variables. Numbers in parentheses display the median and the interquartile range (Q3-Q1). Significant P-values are marked with an asterisk (*). Effect sizes (r) were calculated using the formula $r = z / \sqrt{n}$, where 'n' is the number of observations, in this case 2 x 122 = 244. Thresholds used for qualitative descriptions of effect size were: zero or nearly zero effect, $0 < |r| \leq 0.1$; small, $0.1 < |r| \leq 0.3$; moderate, $0.3 < |r| \leq 0.5$; large $0.5 < |r| \leq 0.7$; and very large, $|r| > 0.7$. Data were obtained through an online survey among professional clients (n = 244) of Dutch pest controllers. Importance could be indicated on a 1 (not important) to 10 (very important) continuous rating scale.

Table S8 Exact *P* values and effect sizes for importance of animal welfare between animal categories in *subgroup agri*

	Rats and mice as pests	Laboratory animals	Other wild animals	Farm animals	Companion animals
**Rats and mice as pests**		0.5859	0.5880	0.6079	0.6045
**Laboratory animals**	<0.0000005*		0.0136	0.4686	0.4710
**Other wild animals**	<0.0000005*	0.862011		0.5264	0.5251
**Farm animals**	<0.0000005*	<0.0000005*	<0.0000005*		0.0571
**Companion animals**	<0.0000005*	<0.0000005*	<0.0000005*	0.465703	

Table S8: Exact (2-tailed) P-values (under de diagonal line) and absolute effect sizes (above the diagonal line) for differences in the importance of animal welfare for five different animal categories. Exact (2-tailed) P-values were calculated for each pair of methods with the Wilcoxon matched-pairs signed ranks test for 2 dependent variables. Significant P-values are marked with an asterisk (*). Effect sizes (r) were calculated using the formula $r = z / \sqrt{n}$, where 'n' is the number of observations, in this case 2 x 84 = 168. Thresholds used for qualitative descriptions of effect size were: zero or nearly zero effect, $0 < |r| \leq 0.1$; small, $0.1 < |r| \leq 0.3$; moderate, $0.3 < |r| \leq 0.5$; large $0.5 < |r| \leq 0.7$; and very large, $|r| > 0.7$. Data were obtained through an online survey among professional clients (n = 122) of Dutch pest controllers. In this table only the results from *subgroup agri* (n = 84) are shown. Importance could be indicated on a 1 (not important) to 10 (very important) continuous rating scale.

Table S9 Exact P-values and effect sizes for importance of animal welfare between animal categories in *subgroup other*

	Rats and mice as pests	Laboratory animals	Other wild animals	Farm animals	Companion animals
**Rats and mice as pests**		0.4024	0.4657	0.5304	0.4951
**Laboratory animals**	0.000169*		0.1757	0.3830	0.3423
**Other wild animals**	0.000003*	0.130083		0.3347	0.2218
**Farm animals**	<0.0000005*	0.000183*	0.00219		0.0045
**Companion animals**	0.0000007*	0.001419	0.053043	0.984375	

Exact (2-tailed) P-values (under de diagonal line) and absolute effect sizes (above the diagonal line) for differences in the importance of animal welfare for five different animal categories. Exact (2-tailed) P-values were calculated for each pair of methods with the Wilcoxon matched-pairs signed ranks test for 2 dependent variables. Significant P-values are marked with an asterisk (*). Effect sizes (r) were calculated using the formula $r = z / \sqrt{n}$, where 'n' is the number of observations, in this case 2 x 38 = 76. Thresholds used for qualitative descriptions of effect size were: zero or nearly zero effect, $0 < |r| \leq 0.1$; small, $0.1 < |r| \leq 0.3$; moderate, $0.3 < |r| \leq 0.5$; large $0.5 < |r| \leq 0.7$; and very large, $|r| > 0.7$. Data were obtained through an online survey among professional clients (n = 122) of Dutch pest controllers. In this table only the results from *subgroup other* (n = 38) are shown. Importance could be indicated on a 1 (not important) to 10 (very important) continuous rating scale.

Table S10 Exact P-values and effect sizes for scored welfare impact of control methods in the total group of respondents

	Glue board (8; 10-2.9)	Trap and drown (7; 9.9-2.7)	Trap and release (3; 8-1)	EKO1000 (3; 7.9-1)	Snap trap (2.9; 6.3-1)	Rodenticides (2.8; 6.2-1)	Cat, dog, ferret (2; 5.9-1)	CO2 trap (1.4; 5-1)	Shoot (1.2; 5.4-1)	Preventive methods (1; 2.6-1)
**Glue board (8; 10-2.9)**		0.0749	0.2512	0.3645	0.4463	0.4330	0.4145	0.4781	0.4627	0.3956
**Trap and drown (7; 9.9-2.7)**	0.244413		0.2180	0.3841	0.3971	0.3551	0.4073	0.4572	0.4521	0.3940
**Trap and release (3; 8-1)**	0.000064	0.000563		0.0031	0.0730	0.0505	0.1003	0.1684	0.1448	0.2845
**EKO1000 (3; 7.9-1)**	<0.0000005*	<0.0000005*	0.962106		0.1208	0.1077	0.1545	0.2772	0.2686	0.2394
**Snap trap (2.9; 6.3-1)**	<0.0000005*	<0.0000005*	0.25575	0.059091		0.0568	0.0590	0.1574	0.1519	0.1927
**Rodenticides (2.8; 6.2-1)**	<0.0000005*	<0.0000005*	0.432037	0.092777	0.378646		0.1065	0.2079	0.1982	0.2103
**Cat, dog, ferret (2; 5.9-1)**	<0.0000005*	<0.0000005*	0.117592	0.015284	0.359403	0.096623		0.0897	0.0798	0.1644
**CO2 trap (1.4; 5-1)**	<0.0000005*	<0.0000005*	0.008135	0.000006*	0.013309	0.000894	0.162654		0.0009	0.1039
**Shoot (1.2; 5.4-1)**	<0.0000005*	<0.0000005*	0.02329	0.000012*	0.016993	0.001663	0.214444	0.990276		0.1067
**Preventive methods (1; 2.6-1)**	<0.0000005*	<0.0000005*	0.000004*	0.000136	0.002342	0.000864	0.009762	0.105083	0.096057	

Table S10: Exact (2-tailed) P-values (under de diagonal line) and absolute effect sizes (above the diagonal line) for differences in the scored welfare impact of methods for the control of rats (*Rattus rattus* and *Rattus norvegicus*) and mice (*Mus musculus*). Exact (2-tailed) P-values were calculated for each pair of methods with the Wilcoxon matched-pairs signed ranks test for 2 dependent variables. Numbers in parentheses display the median and the interquartile range (Q3-Q1). Significant P-values are marked with an asterisk (*). Effect sizes (r) were calculated using the formula $r = z / \sqrt{n}$, where 'n' is the number of observations, in this case 2 x 122 = 244. Thresholds used for qualitative descriptions of effect size were: zero or nearly zero effect, $0 < |r| \leq 0.1$; small, $0.1 < |r| \leq 0.3$; moderate, $0.3 < |r| \leq 0.5$; large $0.5 < |r| \leq 0.7$; and very large, $|r| > 0.7$. Data were obtained through an online survey among professional clients (n = 122) of Dutch pest controllers. Welfare impact could be scored on a 1 (no impact) to 10 (very large impact) continuous rating scale.

Table S11 Medians, Inter Quartile Ranges (Q3-Q1), exact P-values and effect sizes for the difference in scored welfare impact of different control methods between *subgroup agri* and *subgroup other*

	Subgroup agri Median (Q3-Q1)	Subgroup other Median (Q3-Q1)	Exact *p* value (2-sided)	Effect size |r|
**Glue board**	6 (9.1-1.8)	10 (10-8)	0.000024*	0.3743
**Trap and drown**	6.1 (8.8-1.1)	10 (10-7.4)	0.000034*	0.3676
**Trap and release**	3.8 (9.1-1)	2 (6-1)	0.23109	0.1088
**EKO1000**	2 (5.9-1)	8 (10-3)	<0.0000005*	0.4321
**Snap trap**	1.1 (4.6-1)	6.1 (8.2-4)	<0.0000005*	0.4303
**Rodenticides**	1.3 (4-1)	7 (10-5)	<0.0000005*	0.5200
**Cat, dog, ferret**	1.2 (5-1)	5 (8-2)	0.000369*	0.3181
**CO2 trap**	1 (2.3-1)	5.3 (8.5-2)	<0.0000005*	0.4931
**Shoot**	1 (3.9-1)	6 (8-2)	0.000001*	0.4287
**Preventive methods**	1.1 (3.9-1)	1 (2.3-1)	0.921181	0.0091

Table S11: Median, Inter Quartile Ranges (IQR, Q3-Q1), exact (2-tailed) P-values (under de diagonal line) and absolute effect sizes (above the diagonal line) for the difference in scored welfare impact of different control methods between *subgroup agri* (n = 84) and *subgroup other* (n = 38). The impact could be indicated on a 1 (no impact) to 10 (very large impact) continuous rating scale. Data were obtained through an online survey among professional clients (n = 122) of Dutch pest controllers. Exact (2-tailed) P-values were calculated for each statement with the Wilcoxon–Mann–Whitney test for independent variables. Significant P-values are marked with an asterisk (*). Effect sizes (r) were calculated using the formula $r = z / \sqrt{n}$, where ‘n’ is the number of observations, in this case 122. Thresholds used for qualitative descriptions of effect size were: zero or nearly zero effect, $0 < |r| \leq 0.1$; small, $0.1 < |r| \leq 0.3$; moderate, $0.3 < |r| \leq 0.5$; large $0.5 < |r| \leq 0.7$; and very large, $|r| > 0.7$.

Table S12 Exact P-values and effect sizes for scored welfare impact of control methods in *subgroup agri*

	Glue board	Trap and drown	Trap and release	EKO1000	Snap trap	Rodenticides	Cat, dog, ferret	CO2 trap	Shoot	Preventive methods
**Glue board**		0.0625	0.1316	0.3760	0.4596	0.4497	0.3971	0.4864	0.4619	0.3154
**Trap and drown**	0.421635		0.1034	0.4065	0.4211	0.3966	0.3951	0.4749	0.4478	0.3280
**Trap and release**	0.088393	0.181719		0.1902	0.2413	0.2474	0.2229	0.3550	0.3096	0.2904
**EKO1000**	<0.0000005*	<0.0000005*	0.013037		0.0875	0.1333	0.0814	0.2845	0.2403	0.1074
**Snap trap**	<0.0000005*	<0.0000005*	0.001463	0.260146		0.0328	0.0062	0.2216	0.1835	0.0365
**Rodenticides**	<0.0000005*	<0.0000005*	0.001104	0.084372	0.675862		0.0253	0.1850	0.1567	0.0382
**Cat, dog, ferret**	<0.0000005*	<0.0000005*	0.003441	0.294927	0.938741	0.746662		0.1873	0.1926	0.0462
**CO2 trap**	<0.0000005*	<0.0000005*	0.000001*	0.000124	0.003397	0.015397	0.014294		0.0332	0.0760
**Shoot**	<0.0000005*	<0.0000005*	0.00003	0.001395	0.016383	0.041703	0.011632	0.672687		0.0667
**Preventive methods**	0.000024	0.00001*	0.000105	0.165932	0.639861	0.625038	0.553818	0.328763	0.392771	

Table S12: Exact (2-tailed) P-values (under de diagonal line) and absolute effect sizes (above the diagonal line) for differences in the scored welfare impact of methods for the control of rats (*Rattus rattus* and *Rattus norvegicus*) and mice (*Mus musculus*). Exact (2-tailed) P-values were calculated for each pair of methods with the Wilcoxon matched-pairs signed ranks test for 2 dependent variables. Significant P-values are marked with an asterisk (*). Effect sizes (*r*) were calculated using the formula $r = z / \sqrt{n}$, where 'n' is the number of observations, in this case 2 x 84 = 168. Thresholds used for qualitative descriptions of effect size were: zero or nearly zero effect, $0 < |r| \leq 0.1$; small, $0.1 < |r| \leq 0.3$; moderate, $0.3 < |r| \leq 0.5$; large $0.5 < |r| \leq 0.7$; and very large, $|r| > 0.7$. Data were obtained through an online survey among professional clients (n = 122) of Dutch pest controllers. In this table only the results from *subgroup agri* (n = 84) are shown. Welfare impact could be scored on a 1 (no impact) to 10 (very large impact) continuous rating scale.

Table S13 Exact P-values and effect sizes for scored welfare impact of control methods in *subgroup other*

	Glue board	Trap and drown	Trap and release	EKO1000	Snap trap	Rodenticides	Cat, dog, ferret	CO2 trap	Shoot	Preventive methods
**Glue board**		0.0561	0.2736	0.1926	0.2353	0.2145	0.2456	0.2570	0.2619	0.3060
**Trap and drown**	0.396866		0.2563	0.1795	0.1917	0.1350	0.2444	0.2320	0.2534	0.2921
**Trap and release**	0.000003*	0.000017*		0.2205	0.1946	0.2344	0.1300	0.1431	0.1544	0.1456
**EKO1000**	0.001542	0.003479	0.000294		0.1185	0.0227	0.1616	0.1422	0.1702	0.2652
**Snap trap**	0.000084	0.00201	0.001687	0.064446		0.1134	0.0895	0.0250	0.0697	0.2712
**Rodenticides**	0.000342	0.033895	0.000108	0.735176	0.077689		0.1699	0.1493	0.1636	0.2786
**Cat, dog, ferret**	0.000036	0.000038	0.041609	0.010148	0.166658	0.006641		0.0554	0.0531	0.2141
**CO2 trap**	0.000008*	0.000084	0.024335	0.024414	0.706054	0.018219	0.395985		0.0271	0.2365
**Shoot**	0.000005*	0.000014*	0.01447	0.005932	0.28574	0.008721	0.417591	0.684047		0.2237
**Preventive methods**	<0.0000005*	0.0000004*	0.021534	0.000005*	0.000003*	0.000001*	0.000459	0.000078	0.00021	

Table S13: Exact (2-tailed) P-values (under de diagonal line) and absolute effect sizes (above the diagonal line) for differences in the scored welfare impact of methods for the control of rats (*Rattus rattus* and *Rattus norvegicus*) and mice (*Mus musculus*). Exact (2-tailed) P-values were calculated for each pair of methods with the Wilcoxon matched-pairs signed ranks test for 2 dependent variables. Significant P-values are marked with an asterisk (*). Effect sizes (r) were calculated using the formula $r = z / \sqrt{n}$, where 'n' is the number of observations, in this case 2 x 38 = 76. Thresholds used for qualitative descriptions of effect size were: zero or nearly zero effect, $0 < |r| \leq 0.1$; small, $0.1 < |r| \leq 0.3$; moderate, $0.3 < |r| \leq 0.5$; large $0.5 < |r| \leq 0.7$; and very large, $|r| > 0.7$. Data were obtained through an online survey among professional clients (n = 122) of Dutch pest controllers. In this table only the results from *subgroup other* (n = 38) are shown. Welfare impact could be scored on a 1 (no impact) to 10 (very large impact) continuous rating scale.

Table S14 Exact P-values and effect sizes for weight of animal interests for different scenarios for the total group of respondents

	Mice in a hospital kitchen (1; 1.2-1)	Mice in a supermarket (1; 1.7-1)	Mice on a pig farm (1; 3.8-1)	Rats in a cow stable (1; 4-1)	Rats on a children's farm (1; 5-1)	Mice in an animal shelter (1.4; 5.2-1)	Rats at a garbage plant (1.4; 6-1)	Rats in the sewers (1.3; 7.1-1)	Rats in a private backyard (1.9; 7.5-1)	Rats in a ditch (4; 8-1)	Mice in a private backyard (5; 8-1)	Rats alons a golf course (5; 8-1)
**Mice in a hospital kitchen (1; 1.2-1)**		0.2379	0.2726	0.3023	0.2707	0.3671	0.3470	0.3708	0.4156	0.4649	0.4730	0.4693
**Mice in a supermarket (1; 1.7-1)**	0.000183		0.1553	0.1972	0.1950	0.3058	0.2829	0.3162	0.3748	0.4320	0.4411	0.4526
**Mice on a pig farm (1; 3.8-1)**	0.000016	0.020842		0.0628	0.0494	0.2241	0.2355	0.3008	0.3044	0.4398	0.4473	0.4363
**Rats in a cow stable (1; 4-1)**	0,000001*	0.002925	0.36248		0.0252	0.1689	0.1899	0.2697	0.2774	0.4139	0.4196	0.4397
**Rats on a children's farm (1; 5-1)**	0.000026	0.003299	0.471072	0.714054		0.1988	0.1689	0.2409	0.3020	0.3881	0.4292	0.4219
**Mice in an animal shelter (1.4; 5.2-1)**	<0.0000005*	0,000001*	0.000685	0.011813	0.002715		0.0388	0.1419	0.1723	0.3144	0.3629	0.3263
**Rats at a garbage plant (1.4; 6-1)**	<0.0000005*	0.000013	0.000338	0.004445	0.011947	0.570512		0.1288	0.0920	0.2934	0.3230	0.3732
**Rats in the sewers (1.3; 7.1-1)**	<0.0000005*	0.0000006*	0,000003*	0.000035	0.000255	0.035625	0.057088		0.0026	0.1886	0.2116	0.2304
**Rats in a private backyard (1.9; 7.5-1)**	<0.0000005*	<0.0000005*	0,000002*	0.000021	0,000003*	0.010252	0.176108	0.971587		0.2048	0.3023	0.2453
**Rats in a ditch (4; 8-1)**	<0.0000005*	<0.0000005*	<0.0000005*	<0.0000005*	<0.0000005*	0,000001*	0,000006*	0.004838	0.002142		0.0404	0.1061
**Mice in a private backyard (5; 8-1)**	<0.0000005*	<0.0000005*	<0.0000005*	<0.0000005*	<0.0000005*	<0.0000005*	0.0000005*	0.001488	0,000002*	0.554601		0.0352
**Rats alons a golf course (5; 8-1)**	<0.0000005*	<0.0000005*	<0.0000005*	<0.0000005*	<0.0000005*	0.0000004*	<0.0000005*	0.000525	0.00021	0.117925	0.606578	

Table S14: Exact (2-tailed) P-values (under de diagonal line) and absolute effect sizes (above the diagonal line) for differences in the weight of animal interests for different real-life scenarios of the control of rats (*Rattus rattus* and *Rattus norvegicus*) and mice (*Mus musculus*). Animal interests were defined as 'living, freedom and welfare'. Exact (2-tailed) P-values were calculated for each pair of methods with the Wilcoxon matched-pairs signed ranks test for 2 dependent variables. Numbers in parentheses display the median and the interquartile range (Q3-Q1). Significant P-values are marked with an asterisk (*). Effect sizes (r) were calculated using the formula $r = z / \sqrt{n}$, where 'n' is the number of observations, in this case 2 x 109 = 218. Thresholds used for qualitative descriptions of effect size were: zero or nearly zero effect, $0 < |r| \leq 0.1$; small, $0.1 < |r| \leq 0.3$; moderate, $0.3 < |r| \leq 0.5$; large $0.5 < |r| \leq 0.7$; and very large, $|r| > 0.7$. Data were obtained through an online survey among professional clients (n = 122) of Dutch pest controllers. Weight of animal interests (e.g., welfare) could be scored on a 1 (animal interests do not weigh) to 10 (animal interests weigh heavily) continuous rating scale.

Table S15 Medians, Inter Quartile Ranges (Q3-Q1), exact P-values and effect sizes for differences in the weight of animal interests for different real-life scenarios between *subgroup agri* and *subgroup other*

	Subgroup agri Median (Q3-Q1)	Subgroup other Median (Q3-Q1)	Exact p value (2-sided)	Effect size |r|
**Mice in a hospital kitchen**	1 (1.1-1)	1.1 (5.35-1)	0.003175	0.2800
**Mice in a supermarket**	1 (1.1-1)	1.8 (6.25-1)	0.0006700	0.3218
**Mice on a pig farm**	1 (1.2-1)	5 (7.2-1)	0.000046*	0.3831
**Rats in a cow stable**	1 (1.3-1)	5.1 (7.05-1)	0,0000008*	0.4608
**Rats on a children's farm**	1 (1.6-1)	4.5 (7-1)	0.0007570	0.3187
**Mice in an animal shelter**	1 (3.4-1)	5.2 (8-2.5)	0.000002*	0.4463
**Rats at a garbage plant**	1 (3.9-1)	6 (8-3.1)	0.0000009*	0.4559
**Rats in the sewers**	1 (4-1)	7.5 (10-4.45)	0.0000008*	0.4588
**Rats in a private backyard**	1.2 (5-1)	6 (8.4-1)	0.006777	0.2578
**Rats in a ditch**	1.7 (5-1)	8.2 (10-6)	<0.0000005*	0.5594
**Mice in a private backyard**	2.4 (6.6-1)	8 (9.3-5)	0.000132	0.3595
**Rats along a golf course**	3.6 (6-1)	8 (10-5)	0.000064*	0.3750

Table S15: Median, Inter Quartile Ranges (IQR, Q3-Q1), exact (2-tailed) P-values (under de diagonal line) and absolute effect sizes (above the diagonal line) for differences between *subgroup agri* (n = 74) and *subgroup other* (n = 35) in the weight of animal interests for various real-life scenarios of the control of rats (*Rattus rattus and Rattus norvegicus*) and mice (*Mus musculus*). Animal interests were defined as 'living, freedom and welfare'. Data were obtained through an online survey among professional clients (n = 122) of Dutch pest controllers. Weight of animal interests (e.g., welfare) could be scored on a 1 (animal interests do not weigh) to 10 (animal interests weigh heavily) continuous rating scale. Exact (2-tailed) P-values were calculated for each situation with the Wilcoxon–Mann–Whitney test for independent variables. Significant P-values are marked with an asterisk (*). Effect sizes (r) were calculated using the formula $r = z / \sqrt{n}$, where 'n' is the number of observations, in this case 109. Thresholds used for qualitative descriptions of effect size were: zero or nearly zero effect, $0 < |r| \leq 0.1$; small, $0.1 < |r| \leq 0.3$; moderate, $0.3 < |r| \leq 0.5$; large $0.5 < |r| \leq 0.7$; and very large, $|r| > 0.7$.

Table S16 Exact P-values and effect sizes for weight of animal interests for different scenarios for *subgroup agri*

	Mice in a hospital kitchen	Mice in a supermarket	Mice on a pig farm	Rats in a cow stable	Rats on a children's farm	Mice in an animal shelter	Rats at a garbage plant	Rats in the sewers	Rats in a private backyard	Rats in a ditch	Mice in a private backyard	Rats alons a golf course
**Mice in a hospital kitchen**		0.1398	0.1609	0.2181	0.2313	0.3082	0.3031	0.3150	0.4013	0.4193	0.4492	0.4651
**Mice in a supermarket**	0.091919		0.0828	0.1523	0.1840	0.2559	0.2632	0.2667	0.3920	0.3778	0.4429	0.4570
**Mice on a pig farm**	0.049885	0.326481		0.0286	0.1513	0.2830	0.2724	0.2414	0.3684	0.4078	0.4484	0.4671
**Rats in a cow stable**	0.00618	0.064804	0.743896		0.0870	0.2219	0.2312	0.2160	0.3563	0.3809	0.4117	0.4572
**Rats on a children's farm**	0.003705	0.023629	0.066018	0.297664		0.1497	0.1274	0.1615	0.3041	0.3215	0.4423	0.4044
**Mice in an animal shelter**	0.000047	0.001124	0.00027	0.005695	0.069145		0.0169	0.0808	0.2186	0.2179	0.3657	0.3237
**Rats at a garbage plant**	0.000086	0.000883	0.000496	0.003845	0.123971	0.842701		0.0689	0.1601	0.2267	0.3294	0.3629
**Rats in the sewers**	0.000036	0.00074	0.002505	0.007396	0.048974	0.331866	0.409208		0.1571	0.1846	0.2832	0.2850
**Rats in a private backyard**	<0.0000005*	<0.0000005*	<0.0000005*	0.000003*	0.000085	0.006773	0.051135	0.05574		0.0310	0.2605	0.2032
**Rats in a ditch**	<0.0000005*	0.0000005*	<0.0000005*	0.0000004*	0.000032	0.007177	0.004985	0.023776	0.7118		0.1590	0.2057
**Mice in a private backyard**	<0.0000005*	<0.0000005*	<0.0000005*	<0.0000005*	<0.0000005*	0.000002*	0.000023	0.000364	0.001029	0.052823		0.0250
**Rats alons a golf course**	<0.0000005*	<0.0000005*	<0.0000005*	<0.0000005*	<0.0000005*	0.000036	0.000002*	0.000349	0.012577	0.011521	0.764312	

Table S14: Exact (2-tailed) P-values (under de diagonal line) and absolute effect sizes (above the diagonal line) for differences in the weight of animal interests for different real-life scenarios of the control of rats (*Rattus rattus* and *Rattus norvegicus*) and mice (*Mus musculus*). Animal interests were defined as 'living, freedom and welfare'. Exact (2-tailed) P-values were calculated for each pair of methods with the Wilcoxon matched-pairs signed ranks test for 2 dependent variables. Significant P-values are marked with an asterisk (*). Effect sizes (r) were calculated using the formula $r = z / \sqrt{n}$, where 'n' is the number of observations, in this case 2 x 74 = 148. Thresholds used for qualitative descriptions of effect size were: zero or nearly zero effect, $0 < |r| \leq 0.1$; small, $0.1 < |r| \leq 0.3$; moderate, $0.3 < |r| \leq 0.5$; large $0.5 < |r| \leq 0.7$; and very large, $|r| > 0.7$. Data were obtained through an online survey among professional clients (n = 122) of Dutch pest controllers. In this table only the results from *subgroup agri* (n = 74) are shown. Weight of animal interests (e.g., welfare) could be scored on a 1 (animal interests do not weigh) to 10 (animal interests weigh heavily) continuous rating scale.

Table S17 Exact P-alues and effect sizes for weight of animal interests for different scenarios for *subgroup other*

	Mice in a hospital kitchen	Mice in a supermarket	Mice on a pig farm	Rats in a cow stable	Rats on a children's farm	Mice in an animal shelter	Rats at a garbage plant	Rats in the sewers	Rats in a private backyard	Rats in a ditch	Mice in a private backyard	Rats alons a golf course
**Mice in a hospital kitchen**		0.3567	0.4088	0.4307	0.3351	0.4723	0.4168	0.4628	0.4459	0.5538	0.5197	0.4798
**Mice in a supermarket**	0.001343		0.2454	0.2657	0.2058	0.4042	0.3178	0.3996	0.3457	0.5353	0.4436	0.4521
**Mice on a pig farm**	0.000153	0.039032		0.1015	0.1019	0.1309	0.1954	0.3934	0.1659	0.5117	0.4440	0.3889
**Rats in a cow stable**	0.000051	0.0245	0.416138		0.2555	0.0794	0.1126	0.3479	0.1108	0.4858	0.4250	0.4102
**Rats on a children's farm**	0.003571	0.087181	0.40672	0.031052		0.2815	0.2398	0.3350	0.3080	0.5231	0.4391	0.4507
**Mice in an animal shelter**	0.000011*	0.000276	0.283409	0.52158	0.016567		0.0837	0.2276	0.0791	0.4959	0.3571	0.3373
**Rats at a garbage plant**	0.000181	0.006323	0.104937	0.357568	0.044261	0.494563		0.2158	0.0354	0.4032	0.3334	0.3871
**Rats in the sewers**	0.000021	0.00041	0.000511	0.002576	0.003921	0.056726	0.071848		0.2455	0.2023	0.0505	0.0874
**Rats in a private backyard**	0.000038	0.002622	0.170215	0.365465	0.008374	0.521403	0.775939	0.039105		0.4953	0.3778	0.3274
**Rats in a ditch**	<0.0000005*	0.0000002*	0.000001*	0.000005*	0.0000003*	0.000002*	0.000311	0.092503	0.000002*		0.2346	0.1095
**Mice in a private backyard**	0.0000006*	0.000065	0.000061	0.000146	0.000085	0.001968	0.00396	0.686562	0.000641	0.049273		0.0686
**Rats alons a golf course**	0.00001*	0.000041	0.000603	0.000253	0.000043	0.003608	0.000366	0.477314	0.004685	0.372555	0.579131	

Table S17: Exact (2-tailed) P-values (under de diagonal line) and absolute effect sizes (above the diagonal line) for differences in the weight of animal interests for different real-life scenarios of the control of rats (*Rattus rattus* and *Rattus norvegicus*) and mice (*Mus musculus*). Animal interests were defined as 'living, freedom and welfare'. Exact (2-tailed) P-values were calculated for each pair of methods with the Wilcoxon matched-pairs signed ranks test for 2 dependent variables. Significant P-values are marked with an asterisk (*). Effect sizes (r) were calculated using the formula $r = z / \sqrt{n}$, where 'n' is the number of observations, in this case 2 x 35 = 70. Thresholds used for qualitative descriptions of effect size were: zero or nearly zero effect, $0 < |r| \leq 0.1$; small, $0.1 < |r| \leq 0.3$; moderate, $0.3 < |r| \leq 0.5$; large $0.5 < |r| \leq 0.7$; and very large, $|r| > 0.7$. Data were obtained through an online survey among professional clients (n = 122) of Dutch pest controllers. In this table only the results from *subgroup other* (n = 35) are shown Weight of animal interests (e.g., welfare) could be scored on a 1 (animal interests do not weigh) to 10 (animal interests weigh heavily) continuous rating scale.
